# Phenotypic and molecular analysis of seabuckthorn accessions reveal promising genotypes and candidate genes associated with micronutrients

**DOI:** 10.1186/s12870-026-09204-3

**Published:** 2026-06-12

**Authors:** Sadia Hakeem, Muhammad Abu Bakar Saddique, Martin Wiehle, Zulfiqar Ali

**Affiliations:** 1 Institute of Plant Breeding and Biotechnology, Faculty of Agriculture and Environmental Sciences, MNS University of Agriculture, Multan, Pakistan; 2 Department of Horticulture, Faculty of Agriculture, Ataturk University, Erzurum, 25240 Turkey; 3 Organic Plant Production and Agroecosystems Research in the Tropics and Subtropics, University of Kassel, Steinstrasse 19, Witzenhausen, D-37213 Germany; 4 Witzenhausen, Germany; 5 Department of Plant Breeding and Genetics, University of Agriculture, Faisalabad, Pakistan

**Keywords:** AKR, Ascorbic acid, Fruit colour, *Hippophae rhamnoides*, Iron, Phytic acid, Zinc

## Abstract

Seabuckthorn (genus *Hippophae* L., family Elaeagnaceae), rich in vitamin C, anthocyanin, and minerals including iron (Fe) and zinc (Zn), is a potential candidate to combat micronutrient malnutrition. In this study, high diversity for Fe (39.7-341.5 mg kg^− 1^), Zn (0.6–12.7 mg kg^− 1^), and vitamin C (40–595 mg 100 g^− 1^) was found in 70 accessions across Gilgit region of northern Pakistan (2444–3172 m.a.s.l). Orange/yellow fruit colours abundantly found at lower altitudes showed the highest Fe concentration (250.03 ± 61.5 mg kg^− 1^). Despite high phytic acid levels (785–1811 mg kg^− 1^), low ratios for PA: Fe < 0.8, PA: Zn < 0.04 suggested high bioavailability. 18 *HrAKR* genes were identified, with 10 confirmed by PCR in the studied accessions. Three *HrAKR*s were consistently expressed, while seven were differentially expressed across tissues suggesting role in nutrient metabolism. Accessions of a higher altitude region (Sost) combined higher micronutrient levels, greater bioavailability, and stronger *AKR* expression. These findings highlight potential of HrAKR-associated traits for improving nutritional value of seabuckthorn through gene pyramiding. Further functional studies are needed to identify the function of each *HrAKR* gene to enable molecular marker development for seabuckthorn breeding programs.

**Supplementary Information:**

The online version contains supplementary material available at 10.1186/s12870-026-09204-3.

## Introduction

Malnutrition remains a leading cause of child mortality despite increased food production [1]. Iron (Fe), zinc (Zn) and vitamin C/L-ascorbic acid (AA) deficiencies are among the most prevalent forms of micronutrient malnutrition worldwide [2]. Moreover, 15% of the people with functional iron deficiency also exhibit vitamin C deficiency [3], suggesting a strong interrelation. Conventional biofortification efforts targets staple crops, but nutrient-dense neglected and underutilized species (NUS) like seabuckthorn remain poorly characterized for their genetic mechanisms underlying micronutrient accumulation [4, 5]. Adapted to marginal lands and disturbed habitats [6], these species can be commercialized without competing with staple crops [7].

Seabuckthorn *(Hippophae rhamnoides*), is considered a “superfood” owing to its rich nutritional profile. The fruits contain 200–1500 mg 100 g^− 1^ of vitamin C (~ 15 times higher than orange fruit [8]), as well as vitamins D, E, K and P [9], fatty acids (mainly sterols, 1.5–3.5% in fruit pulp and 9.9–19.5% in seed [10]), 0.5–25 mg l00 g^− 1^ anthocyanin, and 24 minerals including iron (Fe; 40–150 ppm) [11], and zinc (Zn; 32 ppm) [12], in addition to a minimum 190 bioactive compounds [13]. Contrary to global situation, in Pakistan, it is underutilized and underdeveloped despite a wide range of morphological and genetic diversity [14]. Most research on seabuckthorn has focused on compositional analysis [15], processing [16], and antioxidant profiling [13], with limited attention to the genetic basis of nutrient bioavailability.

Despite their distinct functions in cells, Fe and Zn are controlled by a complex and intertwined network of genes. Interactions between their homeostatic networks stem from shared chelators like ferric chelate reductase (FRO), nicotinamide synthase (NAS), and transporters, for instance iron and zinc transporters (IRT and ZRT), driven by gene families like ZIP (zinc-iron permease) and YSL (Yellow-stripe like) [17]. However, very few families have been reported that are involved in Fe, Zn, and vitamin C simultaneously; for instance aldo-keto-reductase (*AKR*) gene family members regulate both Fe/Zn uptake [18–20] and vitamin C biosynthesis [21, 22]. The *AKR4B (5–8)* genes encode deoxyumugineic acid synthase phytosiderophore responsible for Fe uptake in barley, maize, rice, and wheat, respectively [23]. Similarly, *AKR4B4* encodes d-galacturonate reductase (GalUR) [21] – a key enzyme in the biosynthesis of L-ascorbic acid from d-galacturonic acid [22] in fruits like citrus [24]. Overexpression of this gene enhanced vitamin C concentrations in tomato [25].

The fruit colour is a well-known phenotypic marker for vitamins and minerals in fruits and vegetables. In seabuckthorn, red, orange and yellow colours of berries are due to the anthocyanins, and carotenoids, respectively [26]. Carotenoids are important antioxidants and are generally linked with nutritional quality. For instance, these are indirectly involved in iron uptake and ferritin synthesis (the bioavailable form of iron) [27]. Similarly, vitamin C facilitates ferritin synthesis and stability, thereby enhancing its bioavailability in plants and animals [28]. These interlinked roles suggest a common regulatory mechanism underlying vitamin C accumulation, fruit colour, and micronutrient uptake and biosynthesis - potentially mediated by AKR gene family, but its pathways remain underexplored.

To achieve this, germplasm was characterized for fruit morphological traits (colour, diameter, volume, moisture, weight) and micronutrient concentrations (Fe, Zn). Selected accessions were evaluated for vitamin C and phytic acid to estimate the bioavailability of micronutrients. Lastly, the accessions were checked for the presence/absence of *AKR* genes and their expression in fruit and leaf tissues. This study investigates whether the diversity and expression patterns of *HrAKR* genes in seabuckthorn accessions are associated with variations in iron, zinc and vitamin C accumulation, and bioavailability, and may be indirectly linked to fruit colour. This would provide a genetic basis for the development of nutrient-dense varieties to combat micronutrient malnutrition.

## Materials and methods

### Plant material

**Fig. 1:**
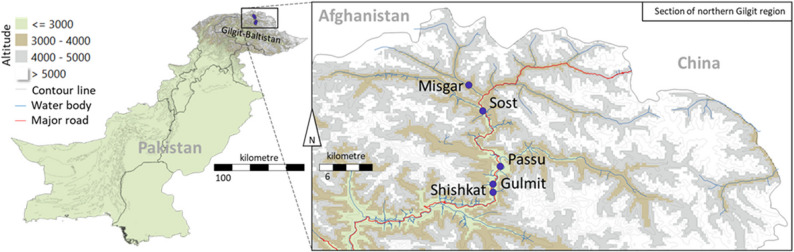
Geographical coordinates of the five seabuckthorn sampling populations in Gilgit, Pakistan

Five locations at different altitudes in the Gilgit region of the northern Karakorum Mountains, Pakistan, were selected for this study (Fig. 1). These sites were chosen to capture maximum environmental heterogeneity including edaphic and microclimatic factors. From each site, fourteen accessions were collected to maintain diversity while keeping a 100 m distance among accessions to avoid clonal sampling and dendrometric traits as well as fruit properties assessed (Table S1). The coordinates and the elevation of each sampling point were determined using a handheld GPS device (Garmin OREGON-750, accuracy up to 2.4 m, GARMIN^®^ Ltd., New Taipei, Taiwan).

### Soil profiling

Soil samples were collected from a depth of 10–30 cm at each location to study the soil physiochemical properties. The analysis targeted soil texture, soil saturation percentage, pH (1:1 soil-to-water ratio), electrical conductivity (EC, mS cm^− 1^), organic matter (OM, %), Fe (mg kg^− 1^), Zn (mg kg^− 1^), potassium (K; mg kg^− 1^), and phosphorus (P; mg kg^− 1^). All analyses were performed following standard soil testing protocols outlined by the International Centre for Agricultural Research in the Dry Areas (ICARDA) [29]. Nutrients were isolated using the diethylenetriaminepentaacetic acid (DTPA) method and quantified using an atomic absorption spectrophotometer (NovAA400P, Analytik Jena GmbH, Jena, Germany). For calibration, certified reference material (CRM 1000 mg l^− 1^) of Fe/Zn was used to prepare working standards of 8, 4, 2, 1, 0.5 and 0.25 ppm through serial dilution method. The calibration curves showed strong linearity (R^2^ > 0.99) (Figure S1 a, b), while the detection limits of the instrument for Fe and Zn were 0.02 ppm for Fe and 0.005 ppm for Zn, according to manufacturer’s instruction.

### Nutrient analysis of fruits for iron, zinc, phytic acid, and ascorbic acid

The Fe and Zn concentrations were measured using the wet digestion method by Ryan et al. [30] and as explained by Hakeem et al. [31]. The digested samples (three biological and technical repeats each) were analysed using an atomic absorption spectrophotometer (NovAA400P, Analytik Jena GmbH, Jena, Germany).

Ten accessions (two from each location) were selected based on contrasting morphological traits especially fruit colour (different classes), and highest and lowest Fe and Zn concentrations from each location, to quantify phytic acid (PA) and AA in berries and to assess the bioavailability of micronutrients. Phytic acid was analyzed using a phytic acid (phytate)/total phosphorus assay kit (Megazyme Ltd., Wicklow, Ireland) following the manufacturer’s instructions. Micronutrient bioavailability was measured using the molar ratio of phytic acid to Fe and Zn. The moles of phytic acid, Fe and Zn were calculated by dividing their respective concentration by their atomic weights. The molar ratios were then determined by dividing moles of phytic acid by moles of respective micronutrients [32]. Vitamin C concentration was determined using the titration method with 2,6-dichlorophenolindophenol (DCPIP) as the dye indicator, following the procedure described by Abdulkadir [33]. Three biological repeats per sample were used, however ten berries were pooled as per required sample weight for each repeat.

### Phenotyping for fruit traits

**Fig. 2:**
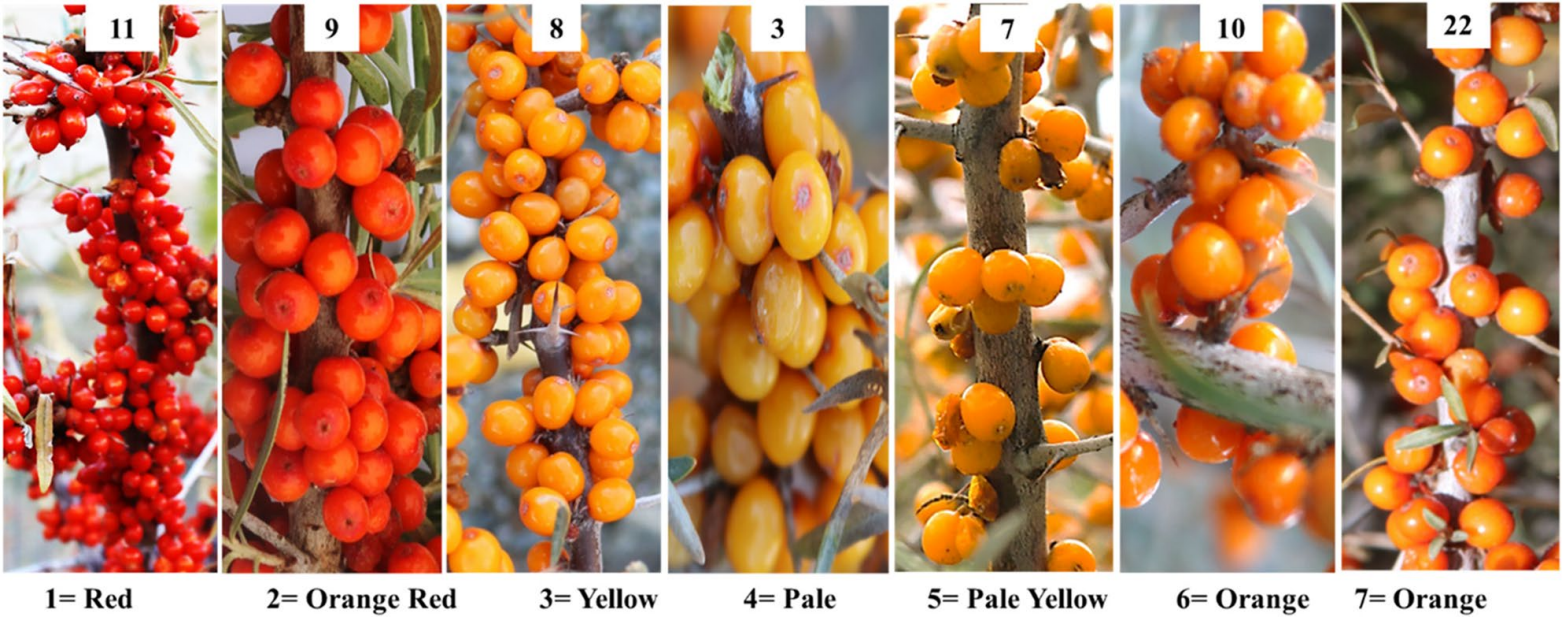
Categories and scale of fruit colour found in the seventy seabuckthorn accessions. Numbers in the white boxes indicate the frequency of accessions for respective colours

The accessions were categorized into seven distinct fruit colour phenotypes visually assessed using a scale between 1 and 7 corresponding red to orange yellow (Fig. 2). The fruit length and width (cm) were measured using a digital vernier calliper (500-196-30, Mitutoyo America Corporation, Aurora, Illinois, United States). The fresh weight (g) of ten fruits per accession was measured, followed by dry weight (g) determination after shade drying the fruits to a constant weight. The fruit moisture content % was measured using the following formula;

$$\:Fruit\:moisture\:\left(\%\right)=\frac{Fresh\:weight-dry\:weight}{fresh\:weight}\times\:100$$

### Identification and retrieval of *AKR* domain containing genes

The protein family database (http://pfam.xfam.org) was used to identify the protein domain of *AKR* (PF00248). The protein sequences of seabuckthorn (*H. rhamnoides*) were retrieved from the *H. rhamnoides* Information Archive (http://hipp.shengxin.ren/DomainSearch.html).

Presence of the *AKR* domain in each sequence was further confirmed through the pfam database. The redundant sequences and the transcripts lacking significant similarity to PF00248 were excluded from further analysis.

For comparative analysis, *AKR* protein sequences from *Arabidopsis thaliana* (model dicot), *Chlamydomonas reinhardtii* (unicellular model alga), and *Fragaria ananasa* (strawberry; model fruit plant) were retrieved from Phytozome v13 (https://phytozome-next.jgi.doe.gov). Gene identifiers were used as gene names for further analysis (Table S2).

### Phylogenetic analysis

For the construction of the phylogenetic tree, the protein sequences from all selected species were aligned using CLUSTALW5. A maximum likelihood tree was then constructed using IQ-TREE [34], applying the best-fit substitution model (JTT + F + G4) following the Bayesian Information Criterion (BIC) [35]. The consistency of phylogeny was validated using the bootstrap method ultrafast value set to 1000 [36]. The resulting tree was visualized in MEGA11 software (www.megasoftware.net/home).

### Conserved motif, domain, and sequence logo analysis

The ARK domain was validated using NCBI’s Conserved Domain Database (CDD, www.ncbi.nlm.nih.gov/Structure/cdd/wrpsb.cgi) and visualized using TBtools (https://bio.tools/tbtools).

The conserved motifs within *HrAKR* proteins were identified using MEME Suite (version 5.4.1; https://meme-suite.org/meme/tools/meme) with default parameters, except that the maximum number of motifs was set to ten. The protein sequences of *H. rhamnoides* were further used to generate a sequence logo using WEBLOGO (weblogo.berkeley.edu/logo.cgi) [37] to highlight conserved amino acid patterns.

### Sub-cellular localization and analysis of protein features

The sub-cellular localization of *HrAKR* proteins was predicted using CELLO v.2.5 (http://cello.life.nctu.edu.tw). The sub-cellular localization signals were further validated using WoLFPSORT (https://wolfpsort.hgc.jp), while the nuclear localization signals were identified using NLSdb database (https://rostlab.org/services/nlsdb).

Key protein features, including coding DNA sequence (CDS) lengths, protein lengths, molecular weight (kDa), theoretical isoelectric point (pI), and grand average of hydropathy value (GRAVY), were computed using the ExPASy-ProtParam tool (https://web.expasy.org/protparam).

### Identification of *HrAKR*s in seabuckthorn accessions

Genomic DNA was extracted from fresh leaf samples following CTAB method [38]. Three technical repeats per sample were used in the process and extracted DNA was pooled for further PCR amplifications. Gene specific full-length primers were designed for the candidate *HrAKR* genes using AmplifX (v2.1.1, Table S3) and ubiquitin gene (*HrUBQ*) as an internal control. PCR reactions were performed in a 15 µl reaction mixture following Maryam et al. [39] using a BioRAD thermal cycler (Bio-Rad Laboratories, Inc., Hercules, California, USA) (s. also supplementary file). A 50 bp GeneRuler DNA ladder (Thermo Fisher Scientific Inc., Waltham, Massachusetts, USA) was used to determine the amplicon size.

### RNA extraction, first strand cDNA synthesis, and semi-quantitative PCR

To further assess the expression pattern, two accessions from each location were selected and the expression of *HrAKR* genes was evaluated in the fruit-leaf (leaf directly attached to fruit) and fruit tissues. Total RNA was extracted from all the samples using the TRIzol^®^ reagent (Invitrogen, USA; Cat#15596026) following the manufacturer’s instructions. RNA integrity was confirmed by electrophoresis on a 1% agarose gel. One µg of good quality RNA was used for first strand cDNA synthesis using RevertAid First Strand cDNA Synthesis Kit (Thermo Fisher Scientific Inc, Waltham, Massachusetts, USA). The targeted genes were then amplified using thermal cycler (BioRAD Inc, California, USA).

### Statistical analysis

The analysis of variance (ANOVA) was performed using the ‘agricoalae’ package [40]. A nested ANOVA was also performed to evaluate differences among location and genotypes nested within location. The variability analysis, heritability estimates, genetic advance, and coefficient of variation (phenotypic, genotypic, and environmental- PCV, GCV, ECV, respectively) were performed using the ‘variability’ package [41] (α = 0.05). The CVs were classified into high (> 20%), medium (10–20%), and low (< 10%) [42] categories. Broad sense heritability (H^2^) – a measure of total variation caused by genetic differences– was classified as high (> 80%), medium (40–80%), and low (< 40%), while genetic advance as a percentage of mean (GAM) – expected improvement in a trait by selecting best individuals – was categorized into high (> 20%), medium (10–20%), and low (< 10%) [43]. Higher the H^2^ and GAM, higher the trait is genetically controlled and selection will be effective.

For the evaluation of genotypic variation, principal component analysis (PCA) was plotted using ‘ggbiplot2’ package in R, with data scaled and centred as per standard procedure [44]. Pearson’s correlation coefficient was calculated using ‘corrplot’ package [45] to assess the relationship between morphological and biochemical traits. The boxplots were generated using R software v. 4.1.2 [65], while all the other graphs were Excel-generated.

## Results

### Nutrient profile of soil

The soil analysis indicated that soils from all locations had a loamy texture, alkaline nature (pH 7.6–8.4), and were moist at field capacity (37–40% soil saturation) (Table 1); however, the higher-altitude regions had a lower availability of Fe, P, and Zn and a high pH (> 8.1). In contrast, the soils from lower altitude regions were more fertile, except Fe (low concentration in Shishkat - Sh).

**Table 1:** Mean (± SD) physico-chemical properties of soil from the sampling regions

	EC	pH	O.M	Available *P*	Available K	Saturation (%)	Zn	Fe
Misgar (M)	3.4 ± 0.8 a	8.3 ± 0.1 a	0.62 ± 0.1	14.9 ± 0.9	197.0 ± 4.1	36.5 ± 1.9 b	3.4 ± 0.8 a	8.3 ± 0.1
Sost (S)	3.0 ± 1.8 a	8.2 ± 0.2 a	0.67 ± 0.1	15.2 ± 0.9	199.5 ± 6.5	38.25 ± 1.3 ab	3.0 ± 1.8 a	8.2 ± 0.2
Passu (P)	1.5 ± 0.2 b	8.4 ± 0.1 a	0.60 ± 0.1	14.5 ± 0.4	194.3 ± 10.0	39.5 ± 1.0 a	1.5 ± 0.2 b	8.4 ± 0.1
Gulmit (G)	2.7 ± 0.6 ab	7.6 ± 0.1 b	0.65 ± 0.1	14.3 ± 1.0	193.0 ± 12.9	39.5 ± 1.0 a	2.7 ± 0.6 ab	7.5 ± 0.1
Shishkat (Sh)	2.2 ± 0.4 ab	7.8 ± 0.2 b	0.60 ± 0.1	15.2 ± 0.8	194.5 ± 2.9	37.5 ± 2.5 ab	2.2 ± 0.4 ab	7.8 ± 0.2

Values are presented as mean ± standard deviation. Different lowercase letters within a column indicate significant differences among sampling regions according to the Least Significant Difference test (*p* < 0.05). Means with the same letter(s) do not differ significantly

*EC* Electrical conductivity (mS cm^− 1^), *P* Phosphorus (mg kg^− 1^), *K* Potassium (mg kg^− 1^), *Zn* zinc (mg kg^− 1^), *Fe* iron (mg kg^− 1^), *O.M* Organic matter (%)

### Genetic variability for fruit quality traits and micronutrients

#### Classification based on fruit colour

The frequency distribution indicated that 31% (22) of the accessions had orange-yellow colour, preceded by red (15%, 11) and orange colour (14%, 10), while only three out of 70 accessions (4%) had pale fruit colour (Fig. 2).

#### Micronutrient concentrations

**Fig. 3:**
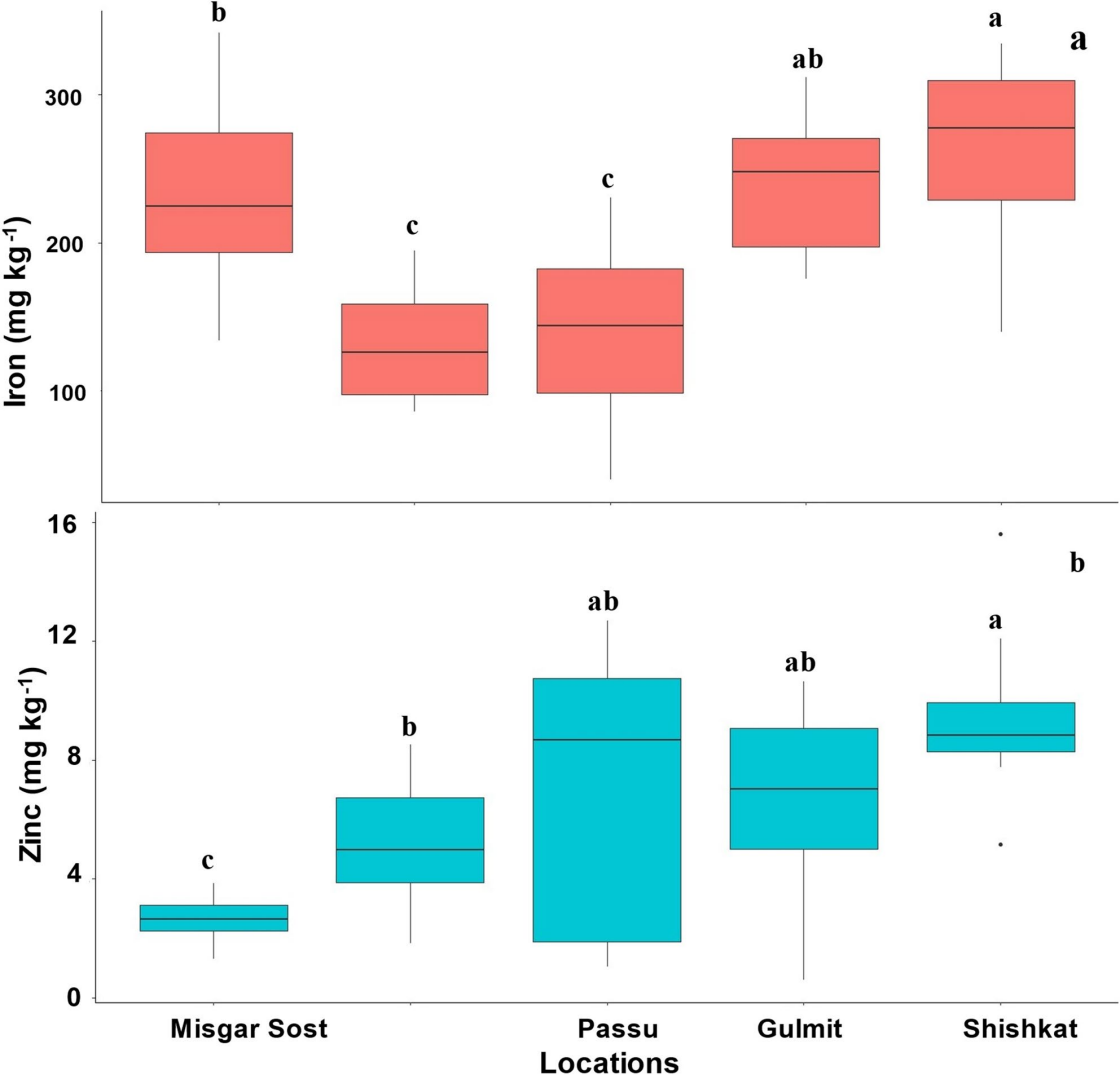
Whisker box plots of iron (**a**) and zinc (**b**) in 70 seabuckthorn accessions at five sampling locations from high (l.) to low (r.) altitude. Different letters above boxplots indicate significant differences among locations according to Fisher's Least Significant Difference (LSD) test at the 5% probability level (p ≤ 0.05). Locations sharing at least one common letter are not significantly different, whereas locations with different letters differ significantly

The Fe concentration among the 70 accessions ranged from 40 mg kg^− 1^ (P2) to 342 mg kg^− 1^ (M3) (Fig. 3a; Figure S2a), while the Zn concentration ranged from 0.6 mg kg^− 1^ (G7) to 12.7 mg kg^− 1^ (P6) (Fig. 3b; Figure S2b). Location-wise, Misgar and Gulmit showed similar trends for Fe. Passu showed largest diversity for Zn concentrations (1–13 mg kg^− 1^) indicated by the broad interquartile range on the box plot, while Misgar had the lowest variability (2.6 ± 0.7 mg kg^− 1^) (Fig. 3b, Figure S2b). Berries from Shishkat had the highest concentrations of Fe (269 mg kg^− 1^), followed by Gulmit (242 mg kg^− 1^) and Misgar (227 mg kg^− 1^). Overall, Shishkat accessions had the highest values for Zn concentrations.

When analysed by fruit colour categories, Fe concentrations were the highest in the orange-yellow berries, preceded by the orange and pale-yellow colours, while lowest was found for the red colour and its shades like orange-red (Table 2).

**Table 2:** Average iron and zinc concentration corresponding to different fruit colours

Colour groups	Iron (mg kg^− 1^)	Zinc (mg kg^− 1^)
Red	171 ± 76	5.5 ± 3.2
Orange Red	163 ± 53	5.3 ± 2.4
Yellow	124 ± 47	5.2 ± 2.1
Pale	184 ± 67	7.5 ± 3.8
Pale Yellow	195 ± 75	6.4 ± 3.1
Orange	215 ± 40	6.0 ± 2.3
Orange Yellow	250 ± 62	6.7 ± 3.5

#### Heritability and genetic variability

Analysis of variance indicated differences (*p < 0.001*) and high variability among accessions for all the traits under study (Table S4). The phenotypic and genotypic coefficient of variations showed similar trends, with phenotypic coefficient of variation (PCV) ranging from 4.6% (fruit moisture; FM) to 49.3% (fruit colour; FC) and genotypic coefficient of variation (GCV) from 3.4% (FM) to 56.5% (Zn) (Table 3). Both PCV and GCV were high for all the traits except those related to fruit size and moisture (i.e., fruit length-FL, fruit diameter-FD, fresh weight-FW, FM). The environmental coefficient of variation was low except for fruit-set (FS), FC, and dry fruit weight (DW). The H^2^ was high for the micronutrients (Fe and Zn), FL, FW, and medium for the rest of the traits. Similarly, GAM was high for all the traits except for FM, which recorded a low value of 5.3%.

**Table 3:** Estimation of genetic parameters in micronutrients and fruit quality traits of 70 seabuckthorn accessions

Traits	X	Max	Min	SE	LSD	σ^2^ e	σ^2^g	σ^2^*p*	ECV	GCV	PCV	H^2^	GA	GAM
Fe	204.0	364.1	39.7	7.2	26.6	155.8	5062.4	5218.2	6.1	34.9	35.4	0.97	144.4	70.8
Zn	5.7	12.7	0.6	0.3	1.2	0.3	10.5	10.8	10.0	56.5	57.4	0.97	6.6	114.6
FC	4.4	7.0	1.0	0.6	2.3	1.2	3.4	4.6	25.2	42.4	49.3	0.74	3.3	75.1
FS	54.2	95.0	10.0	6.1	22.4	110.7	166.5	277.3	19.4	23.8	30.7	0.60	20.6	38.0
FL	6.3	8.8	4.1	0.2	0.8	0.1	0.8	0.9	5.7	14.1	15.2	0.86	1.7	26.9
FD	5.3	7.0	3.6	0.2	0.8	0.1	0.5	0.6	7.0	13.1	14.8	0.78	1.3	23.7
FV	7.4	9.8	5.0	0.3	1.1	0.3	0.9	1.2	7.0	13.1	14.8	0.78	1.7	23.7
FM	87.1	95.4	73.9	1.6	5.7	7.2	9.0	16.1	3.1	3.4	4.6	0.56	4.6	5.3
FW	2.3	4.0	1.0	0.1	0.4	0.0	0.3	0.4	8.1	24.7	25.9	0.90	1.1	48.3
DW	0.3	0.7	0.1	0.0	0.1	0.0	0.0	0.0	20.6	22.6	30.5	0.55	0.1	34.4

*X* Mean, *SE* Standard error, *LSD* Least significant difference (*p ≤ 0.01*), *σ*^*2*^ *e* Environmental variance, *σ*^*2*^ *g* Genotypic variance, *σ*^*2*^ *p* Phenotypic variance, *ECV* Environmental coefficient of variation, *GCV* Genotypic coefficient of variation, *PCV* Phenotypic coefficient of variation, *H*^*2*^ Heritability (broad sense), *GA* Genetic advance, *GAM* Genetic advance as percentage of mean, *Fe* Iron, *Zn* Zinc, *FC* Fruit colour, *FS* Fruit setting percentage, *FL* Fruit length, *FD* Fruit diameter, *FV* Fruit volume, *FM* Fruit moisture, *FW* Fresh weight of fruit, *DW* Dry weight of fruit

#### Selection of accessions based on fruit quality traits across five locations

**Fig. 4:**
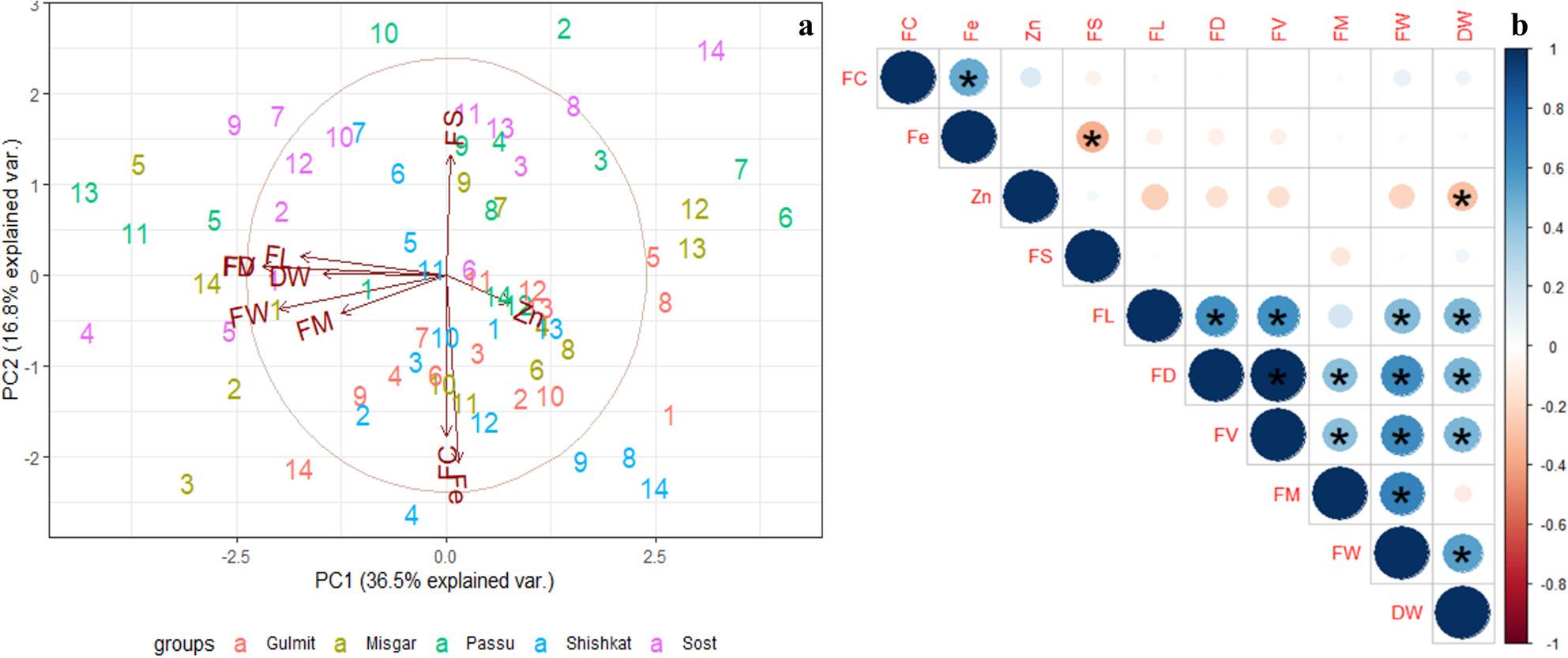
Principal component and Pearson correlation analysis for the morphological and quality traits of fruits for the 70 seabuckthorn accessions across five locations

Overall, the accessions showed a wide range of variability within each location for the FW, DW, FM, and FS. The accessions of Shishkat (low-) and Sost (high-altitude) showed contrasting patterns for Fe, fruit colour (FC), and FS. Accessions from different regions were broadly scattered with no clear grouping by individual traits (Fig. 4a). Iron concentrations were significantly and positively correlated with FC. Zn concentrations showed a negative association with the dry fruit weight (DW). All the fruit morphological traits like fruit length, diameter, volume, moisture, fresh weight and dry weight showed strong positive intercorrelations (Fig. 4b).

#### Ascorbic acid concentrations and bioavailability of iron and zinc in the coreset of accessions

**Fig. 5:**
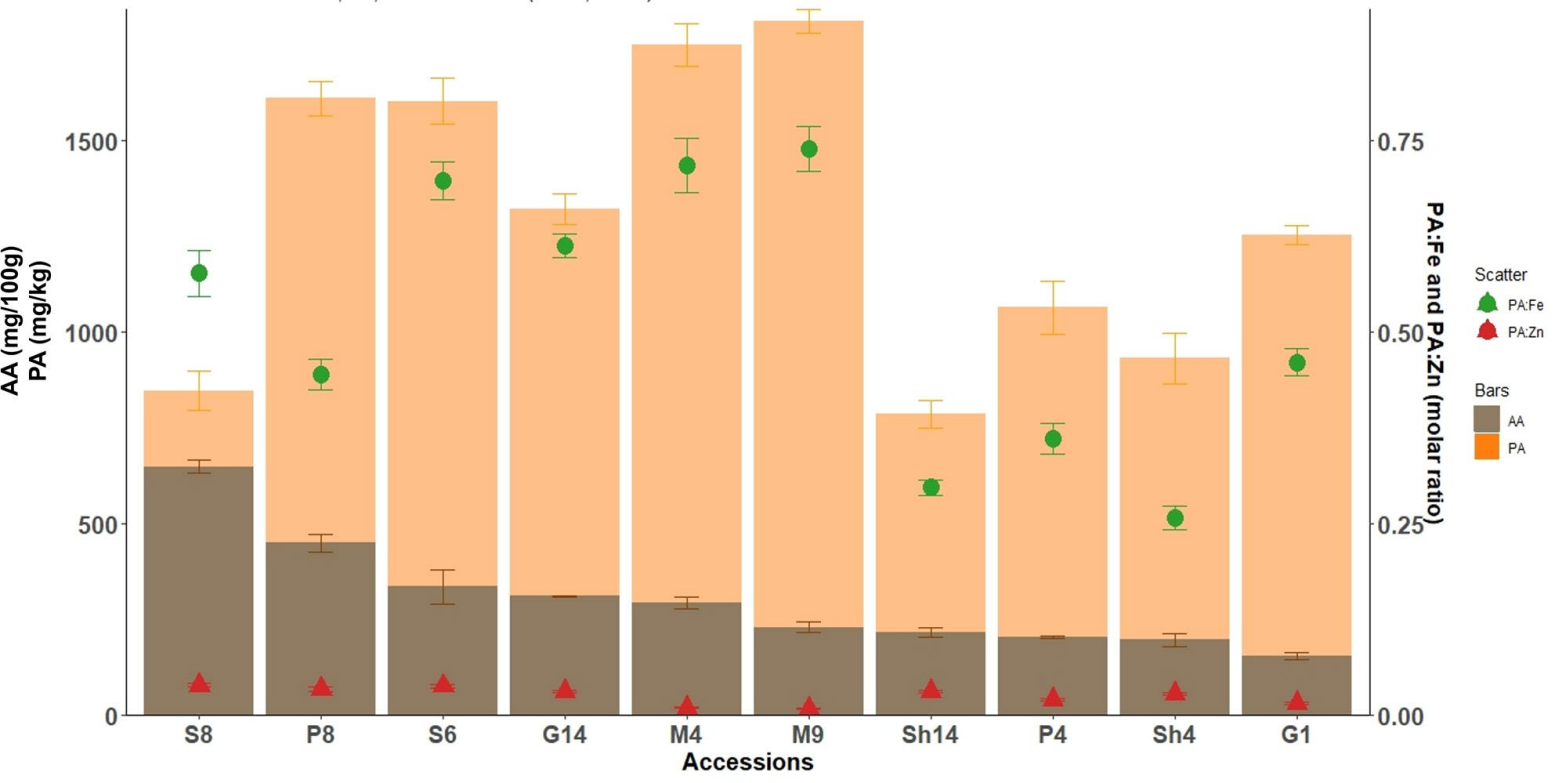
Ascorbic acid, phytic acid, and bioavailability of ten seabuckthorn accessions in relation to phytic acid, iron, and Zn molar ratios. PA: Phytic acid, Fe: Iron, Zn: Zinc, AA: Ascorbic acid

Phytic acid concentration varied widely among accessions, ranging from 785 mg kg^− 1^ (Sh14) to 1811 mg kg^− 1^ in M9 (Fig. 5). While AA concentrations ranged from 155 mg 100 g^− 1^ (G1) to 649 mg 100 g^− 1^ (S8). The accessions of higher altitudes (e.g., M4 and M9) had higher values for the phytic acid content in contrast to low altitudes (e.g. Shishkat, Sh4 and Sh14). A similar trend was found for molar ratios of PA: Fe, followed by accessions from Passu (P4 and P8) and Gulmit (G1). In contrast, the molar ratios of PA: Zn were highest in S8 while lowest in the Misgar accessions (M4 and M9). Overall, PA: Fe ratio was less than 0.8, and PA: Zn was less than 0.04 in all the accessions.

#### Identification and phylogenetic relationships of *HrAKR*s

**Fig. 6:**
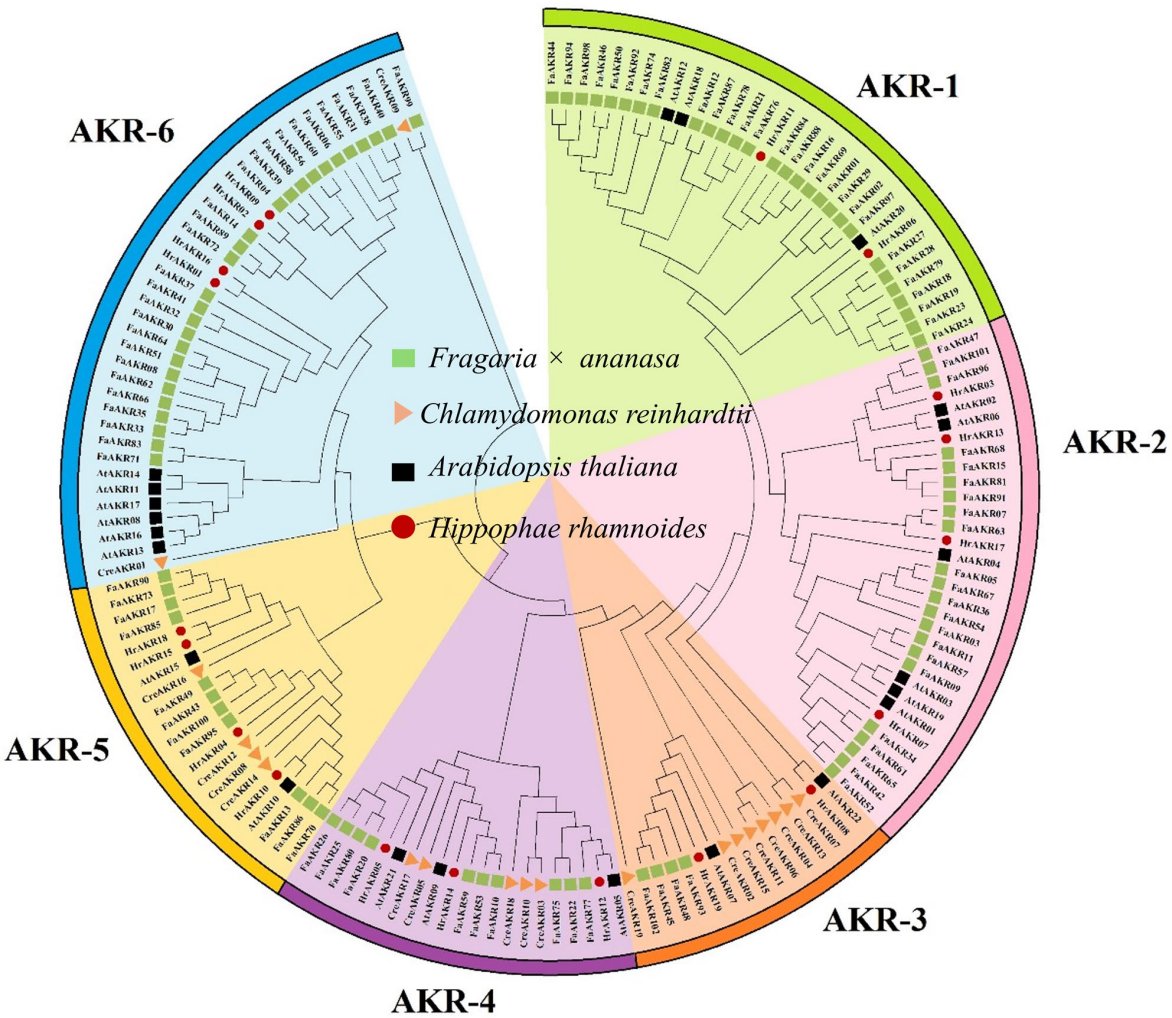
Phylogenetic tree of *Hippophae rhamnoides*, *Arabidopsis thaliana*,* Fragaria ananasa *and *Chlamydomonas reinhardtii AKR* members

A total of 19 *HrAKR* domain containing genes were identified in *H. rhamnoides*, of which 18 were non-redundant and unique and were designated as *HrAKR1* to *HrAKR 18*. In *Fragaria ananasa*, 102 *AKR* genes were identified * (FaAKR1-102)*, 22 in *Arabidopsis thaliana (AtAKR1-22)*, and 19 in *Chlamydomonas reinhardtii (CrAKR1-19)* (Table S2). Phylogenetic analysis grouped all sequences into six major clusters (AKR-1 to AKR-6, Fig. 6). The members of *H. rhamnoides* were distributed among all six groups. Groups 2, 5, and 6 had 4 *HrAKR* members each, followed by group AKR-4 having 3 genes, while AKR-3 and -1 had 2 members each.

#### Subcellular localization and physicochemical properties of the *HrAKR* proteins

**Fig. 7:**
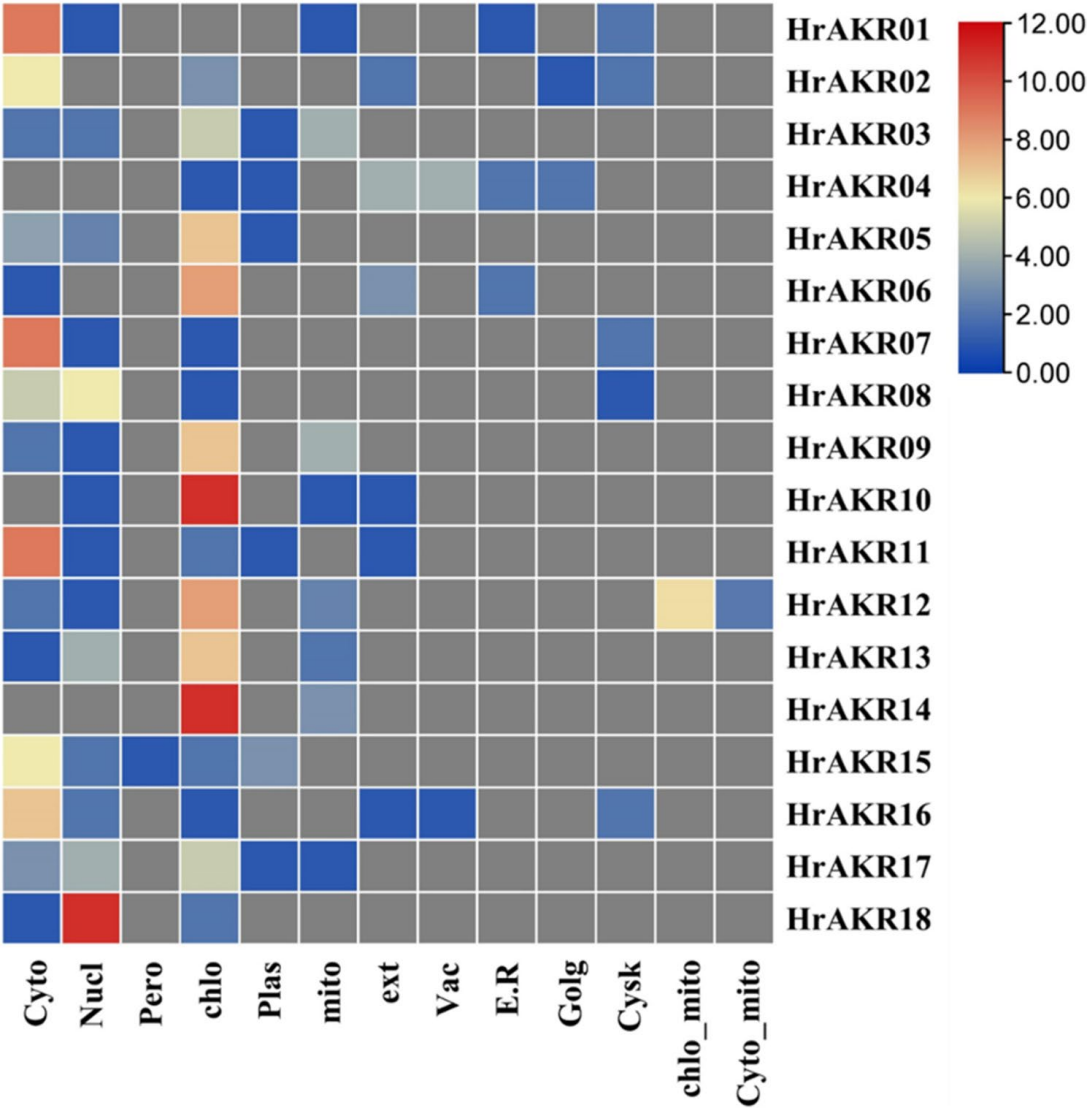
In-silico prediction of sub-cellular localization signal of *HrAKR*s across the cell. Higher to lower scores are indicated by red to blue colour, while grey colour means no score value. Cyto: Cytoplasm, Nucl: Nucleus, Pero: Peroxisomes, Chloro: Chloroplast, mito: Mitochondria, extra: Extracellular, Vac: Vacuole, E.R: Endoplasmic reticulum, Golg: Golgi bodies, Cysk: Cytoskeleton, Plas: Plasma membrane

The predicted subcellular localization of *HrAKR* proteins revealed diverse organelles-specific signals, with higher prediction scores indicating greater confidence in localizing signals (Fig. 7). Cytoplasmic localization was the most frequently predicted, with proteins such as *HrAKR01*, 07, 11, 15 and 16 having high confidence values (≥ 6). Nuclear localization was predominantly observed for *HrARK08* and 18, while chloroplast localization with high prediction score was identified in *HrARK06*, 10, 12 and 14. Notably, *HrAKR12* exhibited dual localization signals in both chloroplasts and mitochondria (chlo_mito). Mitochondrial localization was also predicted for *HrAKR03* and *09*. Signals for peroxisomal and cytoskeletal localization were rare, with only *HrAKR15* showing peroxisomal signals. In total, seven *HrAKR*s were localized in the cytoplasm, 4 genes showed dual localization in cytoplasm and chloroplast and one gene each was predicted to localize in mitochondria, plasma membrane and extracellular space (Figure S3).

The physicochemical properties of *HrAKR* genes showed considerable variability. Protein lengths ranged from 121 to 451 amino acids, while corresponding gene lengths ranged from 366 bp (*HrAKR02*) to 2275 bp (*HrAKR04*) (Table S5). The molecular weight varied between 13.4 kDa (*HrAKR02*) to 50.56 kDa (*HrAKR04*).

#### Domain, motif, and conserved protein residue analyses of *HrAKR* genes

**Fig. 8:**
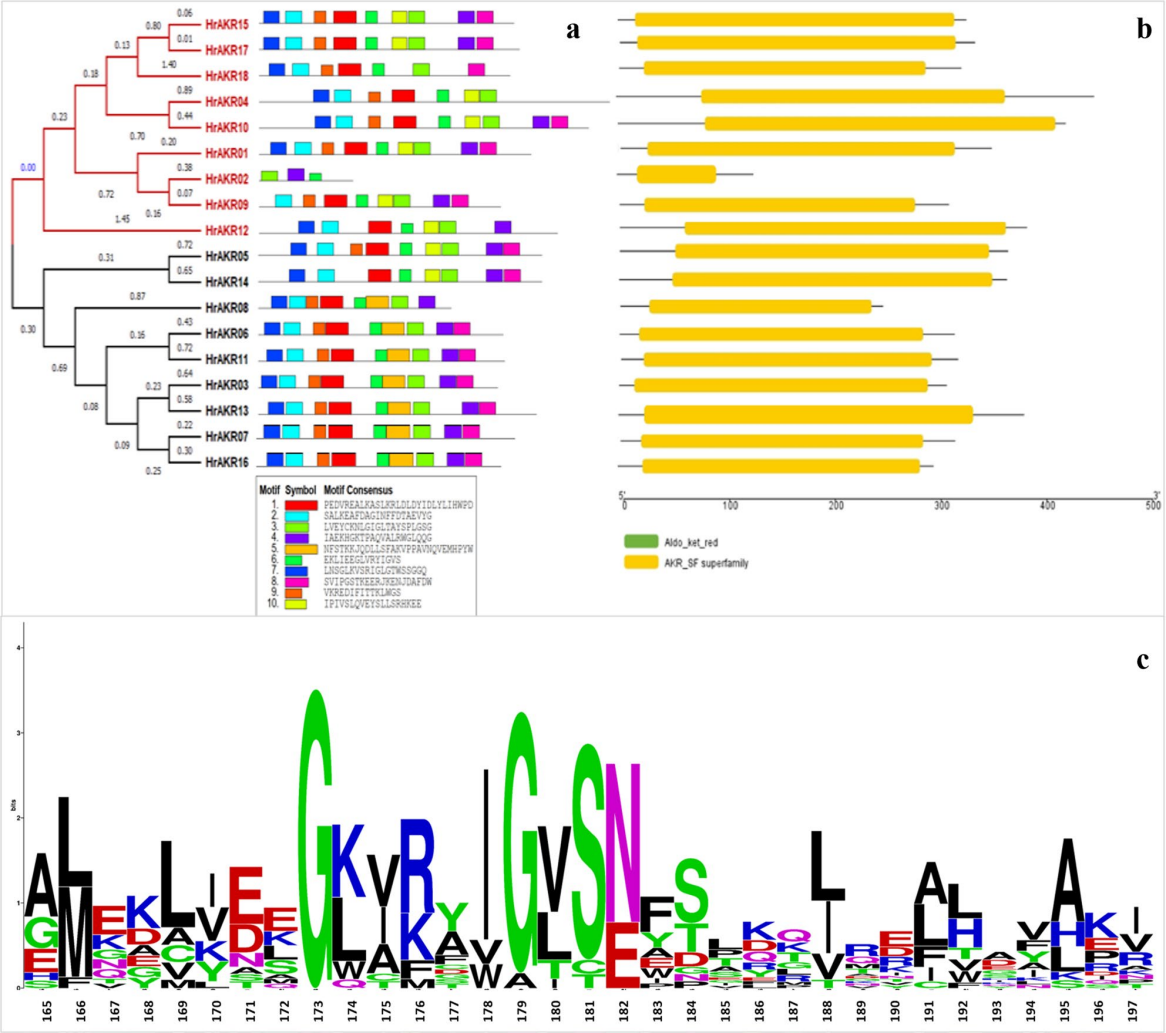
**a**-**c** Motif, domain, and conserved amino acid residue analysis of *HrAKR* proteins. **a** Conserved motifs arranged as per the phylogenetic classification of the *HrAKR*s*. ***b** Conserved domain(s) in *HrAKR*s*.* **c** Sequence logo representation of conserved amino acid residues (165–197) in the *HrAKR* protein family. The height of each letter indicates the relative frequency of amino acids at that position, while overall stack height represents sequence conservation measured in bits.

The *HrAKR* gene family was analyzed to identify conserved protein domains, motifs, and critical amino acid residues, providing critical insight into their potential functions. All *HrAKR* proteins contained only the NADPH-dependent oxidoreductase domain (Fig. 8a). Motif analysis revealed a strong degree of conservation, with all motifs present across most genes except motif 10, which was absent in some members (Fig. 8b). While *HrAKR02* contains only three motifs (motifs 3, 4 and 6). The sequence logo analysis revealed highly conserved residues, including Glycine at positions 173 and 179, Valine (180), Serine (181), and Asparagine (182) (Fig. 8c).

#### Transcript abundance of the *HrAKR*s

**Fig. 9:**
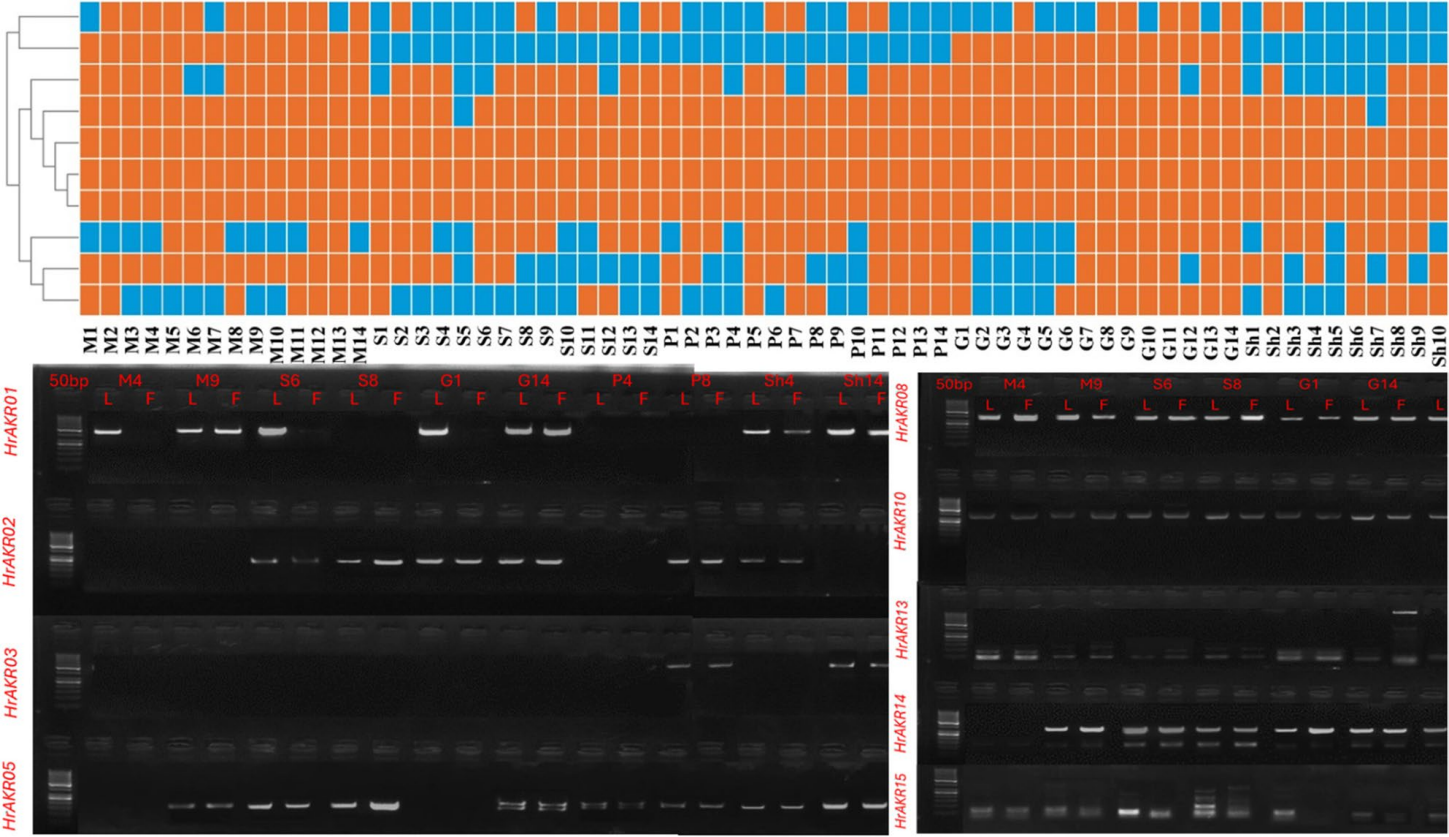
Transcript abundance of *HrAKR* genes in seabuckthorn. **a** Presence/absence of *HrAKR* genes in 70 seabuckthorn accessions. Numbers on the right side are the Gene IDs; orange colour indicates presence while blue colour indicates absence of the gene. **b** Transcripts *HrAKR01*,* 02*,* 03*,* 05*,* 08*,* 10*,* 13*,* 14* and *15* in leaf and fruit tissues. F: Fruit, L: Leaf

Ten out of 18 transcripts were confirmed in 70 accessions (Fig. 9a). *HrAKRs 08*,* 10* and *14* were found in all the accessions. *HrAKR5* was present in all accessions except S5 and Sh7. All other transcripts were differentially present in the accessions. The genes showed differential expressions among accessions, but within an accession, the leaf and fruit tissues showed similar expression patterns (Fig. 9b).

*HrAKR08* showed broad expression across all tissues and accessions. *HrAKR10* and *14* expressed in all accessions except for P10, and M4, respectively. While *HrAKR03* and *15* had low expressions found only in the P8 and Sh14 (*HrAKR03*), and SR1 and PR1 (*HrAKR15*), respectively (Fig. 9b). The *HrAKR05* was absent in M4, G1, and P4, while, *HrAKR02* did not express in M4, M9 (Misgar accessions), P4 and Sh14. *HrAKR13* expressed in Misgar accessions as well as S8, and G14.

## Discussion

Neglected and underutilized species (NUS) are generally semi-domesticated or wild plant species that are well adapted to the local environment but receive limited attention from researchers and farmers. Nonetheless, these species offer tremendous potential to fight poverty, malnutrition, and hunger. The presented study used seabuckthorn to study the biochemistry and gene expressions and found evidence of a close interrelation among assessed variables valuable for breeding and commercial exploitation of seabuckthorn. Empirical evidence was found for the association among orange-yellow fruit colours and iron concentrations based on correlation analysis. High diversity and concentrations for micronutrients were found, with favourable bioavailability levels. The study also indicates that micronutrients and some quality parameters may be governed by additive genes, thus favouring selection for the improvement of these traits. Molecular studies revealed chloroplast-localized genes like *HrAKR10* and *14* expressed ubiquitously, suggesting their possible involvement in photosynthetic redox balancing or metabolite detoxification. Specifically, *HrAKR08* was predicted to localize in both the cytoplasm and nucleus. *HrAKR01* was down-regulated in fruit tissues; *HrAKR03* and *15* only expressed in a few accessions (Fig. 9b). Accession S8 with the highest micronutrients and their bioavailability levels had the highest number of transcripts expressed in both fruit and leaf tissues.

In the present study, significant variation in fruit colour was observed among seabuckthorn accessions from the Gilgit region, identifying seven distinct classes. Yellow and orange-yellow colours collectively accounted for > 45% of the accessions (Fig. 2), predominantly found in Shishkat region. This pattern aligns with findings of Nawaz et al. [14] who reported an increasing frequency of darker fruit shades with high altitude, supporting our finding that lower altitude accessions had a higher frequency of light coloured fruits such as orange and yellow. The orange and yellow fruit colour in seabuckthorn is due to high concentrations of β-carotene (15–25% in berries) [46]. Notably, β-carotene enhances non-heme Fe bioavailability by forming soluble complexes that reduce the inhibitory effects of phytates and polyphenols [27, 47]. A similar connection was found in our study as the iron content was higher in orange-yellow (250 ± 61 mg kg^-1^) followed by orange-coloured fruits (215 ± 40 mg kg^-1^) (Table 1). Association analysis also supported a significant positive correlation between orange/yellow fruit colours and Fe concentrations (Fig. 4b). These findings are consistent with those of Yu et al. [48], who reported that the consumption of orange/yellow fruits enhances dietary intake of both iron and vitamin C content. Our findings suggest that fruit colour can be a visual proxy for selecting germplasm with enhanced nutrients, reducing costs and time in field germplasm explorations. However, as the current study derives this connection based on biological and statistical correlations only, further confirmative studies on molecular basis of this association are needed to establish fruit colour as a phenotypic marker.

Nested analysis of variance indicated significant differences among locations as well as interaction of locations and accessions for all the traits (Table S7). Location and replications interaction was insignificant except for fruit-set, which is expected as plants are grown wildly and prone to animal and human interventions and damage. Altitudinal trends showed that both high-altitude (Misgar) and low-altitude (Shishkat) accessions had higher Fe contents (> 276 mg kg^− 1^), while accessions from intermediate altitudes (Passu) and especially Sost had lower Fe concentration. However, given that the soil Fe and Zn was less available at higher altitudes (Table 1), and soils at lower altitude were more fertile, it might indicate more genotypic influence on nutrient uptake at higher altitudes and edaphic contribution to higher nutrient concentrations at low altitude accessions. Further studies should be conducted to statistically verify this association. This observation partially contrasts with reports by Macek et al. [49], who reported a decreasing trend of nutrient uptake with increasing altitude in the flora of the Himalaya. However, the increased Fe and Zn uptake at high altitudes is reported in other hardy species like Persian ironwood by Kiaei et al. [50], indicating a species-dependent reactions to changing environmental factors. Accessions from higher altitudes like Sost had higher fruit moisture, fruit volume, fresh and dry fruit weight, and fruit diameter (Fig. 4), suggesting an altitudinal influence on both nutritional and morphological traits.

Regardless of the concentrations of micronutrients, their bioavailability largely depends on the presence of antinutritional compounds, particularly phytic acid (PA), polyphenols, and the level of enhancer molecules like vitamin C. The molar ratio of PA: Fe should be less than 1 for significant Fe absorption [51]. For Zn, a molar ratio of < 5, 5–15 and > 15 correspond to 50%, 30% and 15% Zn availability [52], respectively. The recommended dietary allowance (RDA) for vitamin C is 90 mg day^− 1^ for males and 80 mg day^− 1^ for females [53]. Despite high concentrations of phytic acid (Fig. 5), all accessions exhibited ratios below the thresholds (PA: Fe < 1 [51] and PA: Zn < 5 for 50% absorption [52]), suggesting favourable bioavailability of both Fe and Zn in seabuckthorn berries. However, these ratios are indirect measures of bioavailability and given the complex nature of mechanisms of nutrients uptake and biosynthesis of phytic acid and vitamin C, there are several other contributing factors, which should be studied systematically for validation. Vitamin C further contributes to iron uptake and mobilization by enhancing its absorption and translocation to sink tissues. The efflux of AA has been reported to contribute to the reduction of Fe^3+^ to Fe^2+^ in *Arabidopsis* [54]. In current study, AA concentrations varied among accessions, ranging from 155 to 649 mg 100^− 1^. However, accessions with higher AA content such as S8 had lower PA concentrations, implying greater Fe and Zn bioavailability (Fig. 5). Similarly, positive associations were found among Fe concentrations and number of AKRs (Figure S3). These findings are in line with the work of Grillet et al. [54], who found that *Arabidopsis* mutants with low vitamin C content showed reduced iron accumulation in seeds, attributed to their decreased ability to reduce Fe^3+^ to Fe^2+^.

The narrow differences between PCV and GCV for most of the traits suggested limited environmental influence, indicating a predominantly strong genetic control for these traits (Table 3). However, traits such as DW, FM, and FC were moderately influenced by the environment indicated by moderate heritability (0.60, 0.74, respectively) but higher GAM (38, 75%, respectively), and possibility of both additive and non-additive gene effects. These traits could therefore respond to recurrent selection or progeny testing, especially under stabilized environmental conditions. Conversely, traits including FD, FV, FM, and DW had lower heritability (0.55–0.78) and GAM (5–35%) indicating possible influence of non-additive gene action. For improvement of such traits, heterosis breeding or clonal selection may be the suitable approach. High broad-sense heritability estimates, and GAM were observed for Fe (97 and 71%, respectively) and Zn (97 and %, respectively), as well as FC (~ 75%), indicating that these traits may possibly be governed by additive genes and hold strong potential for improvement through selection. However, the contribution of non-additive gene action and environmental influence cannot be neglected due to the lack of narrow-sense heritability estimates. These findings should be validated by narrow sense heritability estimates and across different environmental conditions to rule out the involvement of non-additive genes.

To further explore the genetic basis of nutrient accumulation, we examined the presence/absence of genes and expression profile of *HrAKRs*, with a particular focus on those involved in vitamin C biosynthesis. Ten genes were confirmed while the absence of eight genes may be due to presence/absence variation among accessions, pseudo gene prediction, or sequence polymorphism at the primer binding site [55]. The physicochemical properties of the encoded proteins confirmed genetic divergence (Table S5), suggesting a broad spectrum of functions. All the *HrAKR* proteins had a single NAD(P)H-dependent oxidoreductase domain specific to AKR superfamily (Fig. 8; [63, 64]), consistent with the homologs characterized in *Medicago trunculata* (*MtAKR1-30*) [56], cotton (*Gossypium hirsutum*, *GhAKR40*) [57], and *Brassica rapa* (*BrcAKR22*- a homolog for *GalUR* gene) [57, 58]. The absence of any other domain is indicative of functional specificity across this gene family in seabuckthorn, aligning with other species like *Brassica rapa* [58]. Motif analysis further verified this domain conservation, with all motifs except motif 10 present in most *HrAKRs* (Fig. 7). Interestingly, *HrAKR02* had only three conserved motifs (3, 4, and 6), suggesting functional divergence, gene truncation, or pseudogenization [58], consistent with a homologue in *Brassica rapa* (*BraAKR29*) which also lost most of its motifs [58]. Its corresponding protein was also shorter (13.4 KDa; Table S5). These patterns of conservation and divergence were further validated by sequence logo analysis, with strong conservation of amino acid residues like Gly, Val, and Ser, also observed in other species such as tomato [21, 59]. The flanking regions showed lower conservation, suggesting functional flexibility or potential for diversification.

Subcellular localization analysis indicated that the majority of the *HrAKR* proteins were localized in cytoplasm, chloroplast, and nucleus (Fig. 7), which is consistent with its homologues found in sweet cherry [60]. *HrAKR08* and *18* were localized predominantly in nucleus, suggesting a putative role in redox regulation of nuclear process or gene expression control. While the chloroplast-localized genes like *HrAKR06*, *10*, *12* and *14*, some of which are ubiquitously expressed, maybe involved in photosynthetic redox balancing or metabolite detoxification, as also found in *Medicago truncatula* [56]. *HrAKR12* showed dual localization to chloroplasts and mitochondria - suggesting the possibility of a multifunctional role [61]. Additionally, *HrAKR12* was predicted to contain a nucleus localization signal (NLS) sequence “KRIRPR” (amino acid 28–33), consistent with the localization of the homologue *PaAKR28* in sweet cherry [60]. Conversely, *HrAKR13* contained a nuclear export signal (NES) sequence “LIDLGL” (amino acid 155–160), indicating possible involvement in nucleocytoplasmic transport (Table S6). Only *HrAKR15* was localized in peroxisomes, suggesting a possible role in lipid metabolism or detoxification of reactive oxygen species. This is supported by evidence from Thai Jasmine rice, where *AKR4C14* plays a similar detoxification role [62].

The phylogenetic tree classified 161 *AKR* proteins into six clades, showing a high degree of homology between *H. rhamnoides* and *Arabidopsis thaliana* compared to *Fragaria ananasa* and *Chlamydomonas reinhardtii*. Exceptions included *HrAKR01*, *02*, *09*, and *16*, which clustered more closely to *FaAKR* proteins in clade *AKR-6* (Fig. 6), indicating close evolutionary associations and potential functional similarities [23].

Leaf and fruit tissues showed similar patterns of expression, except for a few transcripts like *HrAKR01* that had reduced or no expression in fruit tissues. *HrAKR03* and *15* showed the lowest expression and were only expressed in a few accessions (Fig. 9b). Interestingly, the accession with the highest concentrations and bioavailability of vitamin C and Fe, such as S8 had the highest number of transcripts expressed in both fruit and leaf tissues (Fig. 9b). However, the gene expression was performed using semi-quantitative methods which compares relative transcript abundance and does not quantify the precise expression. Hence, further validation through quantitative PCR should be coducted. Nonetheless, it was concluded that *HrAKR*s, particularly *HrAKR08* and *14* could be putatively potential molecular targets for improving micronutrient loading in fruit tissues, offering strategic potential for enhancing nutritional quality in seabuckthorn.

## Conclusion

This study provides the first integrated morphological, biochemical and molecular characterization of Fe, Zn, and vitamin C. The strong association between orange-yellow fruit colouration and high Fe concentrations indicate its possible potential as a visual marker for nutrient rich accessions. However, the genetic basis of this statistical correlations should be further studied. Altitudinal variations also influenced micronutrient accumulations with accessions from highest and lowest altitudes having relatively high nutrients compared to the intermediate altitudes. At a molecular level, 18 *HrAKR* genes were identified, with *HrAKR03*, 13 and *15* expressing differentially across accessions, but should be validated by quantitative PCR or functional studies of these genes. The conserved NADPH domain, and chloroplast and cytoplasmic localization of key *HrAKR*s indicate the possibility of involvement in vitamin C biosynthesis, Fe reduction, redox homeostasis, and nutrient uptake, but should be validated by functional studies. Quantitative genetic analyses indicated that Fe, Zn, fruit length and weight are mainly governed by additive gene action and can be improved by direct phenotypic or marker-assisted selection but should be validated by narrow heritability estimates. Fruit set and colour may respond better to recurrent or progeny selection. This integrated analysis revealed that carotenoid-rich fruit colours, low phytic acid content, and high ascorbic acid levels are key markers for enhancing mineral bioavailability, especially iron, in seabuckthorn. These findings establish a practical framework for breeding nutrient-dense cultivars of seabuckthorn contributing to sustainable nutritional security programs.

## Supplementary Information

Supplementary Material 1.

## Data Availability

All data generated or analysed during this study are included in this published article and its supplementary information files. The raw data files and methods are available from the corresponding author upon reasonable request.

## Abbreviations

M: Misgar
S: Sost
P: Passu
G: Gulmit
S: Shishkat
σ^2^ e: Environmental variance
σ^2^ g: Genotypic variance
σ^2^ p: Phenotypic variance
ECV: Environmental coefficient of variation
GCV: Genotypic coefficient of variation
PCV: Phenotypic coefficient of variation
H^2^: Heritability (broad sense)
GA: Genetic advance
GAM: Genetic advance as percentage of mean
Fe: Iron
Zn: Zinc
PA: Phytic acid
AA: Ascorbic acid
FC: Fruit colour
FS: Fruit setting percentage
FL: Fruit length
FD: Fruit diameter
FV: Fruit volume
FM: Fruit moisture
FW: Fresh weight of fruit
DW: Dry weight of fruit
Cyto: Cytoplasm
Nucl: Nucleus
Pero: Peroxisomes
Chloro: Chloroplast
mito: Mitochondria
extra: Extracellular
Vac: Vacuole
E.R: Endoplasmic reticulum
Golg: Golgi bodies
Cysk: Cytoskeleton
Plas: Plasma membrane
NUS: Neglected and underutilized species
