# Supplementary material for: Phenotypic and molecular analysis of seabuckthorn accessions reveal promising genotypes and candidate genes associated with micronutrients
Source: BMC Plant Biol. 2026 Jun 12;26:1025. doi: 10.1186/s12870-026-09204-3 (PMC13263946; doi:10.1186/s12870-026-09204-3)
Supplement: Supplementary file 1 — Supplementary Material 1. [file 12870_2026_9204_MOESM1_ESM.docx]

## Phenotypic and molecular analysis of sea-buckthorn accessions reveal promising genotypes and candidate genes associated with micronutrients

Sadia Hakeem^1,2^, Muhammad Abu Bakar Saddique^1^, Martin Wiehle*^,3,4^, Zulfiqar Ali*^,1,5,6^

^1^Institute of Plant Breeding and Biotechnology, MNS University of Agriculture, Multan, Pakistan

^2^Cotton Research Institute Multan, Ayub Agricultural Research Institute, Faisalabad, Pakistan

^3^Organic Plant Production and Agroecosystems Research in the Tropics and Subtropics, University of Kassel, Steinstrasse 19, D-37213 Witzenhausen, Germany

^4^Centre for International Rural Development, University of Kassel, Steinstrasse 19, D-37213 Witzenhausen, Germany

^5^Department of Plant Breeding and Genetics, University of Agriculture, Faisalabad, Pakistan

^6^Programs Department, Islamic Organization for Food Security, Mangilik Yel Ave. 55/21 AIFC, Unit 4, C4.2, Astana, Republic of Kazakhstan

* Corresponding author: [zulfiqarpbg@hotmail.com](mailto:zulfiqarpbg@hotmail.com), [wiehle@uni-kassel.de](mailto:wiehle@uni-kassel.de)

Table S1. Dendrometric and fruit traits for all the accessions under study

|  |  |  |  | Canopy area (cm) | |  |  | |
| --- | --- | --- | --- | --- | --- | --- | --- | --- |
| Location | Sample Code | Height (cm) | GBH (cm) | WE | NS | No. of trunks | Fruit color | Fruit set (%) |
| Misgar | M1 | 291 | 12 | 420 | 150 | 4 | OY | 70 |
|  | M2 | 300 | 20 | 210 | 171 | 1 | Y | 60 |
|  | M3 | 150 | 4 | 219 | 204 | 4 | OY | 30 |
|  | M4 | 225 | 1.1 | 570 | 150 | 1 | O | 50 |
|  | M5 | 285 | 13 | 360 | 453 | 1 | OR | 35 |
|  | M6 | 420 | 7 | 570 | 390 | 4 | OY | 55 |
|  | M7 | 321 | 1.2 | 363 | 312 | 1 | OR | 50 |
|  | M8 | 285 | 7 | 210 | 231 | 1 | OR | 35 |
|  | M9 | 225 | 9 | 177 | 144 | 1 | R | 85 |
|  | M10 | 198 | 10 | 207 | 198 | 1 | OY | 30 |
|  | M11 | 210 | 5 | 135 | 93 | 1 | OY | 40 |
|  | M12 | 204 | 6 | 219 | 105 | 2 | OY | 55 |
|  | M13 | 249 | 5.6 | 300 | 246 | 1 | Y | 60 |
|  | M14 | 183 | 4 | 99 | 129 | 3 | OY | 20 |
| Sost | S1 | 255 | 6 | 210 | 264 | 1 | YO | 70 |
|  | S2 | 153 | 6 | 165 | 123 | 1 | YO | 60 |
|  | S3 | 120 | 10 | 129 | 180 | 3 | R | 57 |
|  | S4 | 228 | 12 | 201 | 207 | 3 | OY | 45 |
|  | S5 | 150 | 14 | 120 | 132 | 5 | YO | 35 |
|  | S6 | 165 | 11 | 135 | 153 | 3 | YO | 45 |
|  | S7 | 144 | 10.2 | 123 | 189 | 2 | OY | 90 |
|  | S8 | 219 | 5 | 156 | 189 | 1 | O | 75 |
|  | S9 | 225 | 9 | 225 | 225 | 1 | OY | 65 |
|  | S10 | 195 | 7 | 228 | 240 | 1 | OY | 70 |
|  | S11 | 249 | 8 | 240 | 153 | 1 | OY | 72 |
|  | S12 | 213 | 7 | 207 | 195 | 1 | OY | 35 |
|  | S13 | 174 | 5.1 | 129 | 141 | 1 | Y | 60 |
|  | S14 | 135 | 4.5 | 180 | 177 | 1 | OR | 70 |
| Passu | P1 | 102 | 18 | 168 | 192 | 5 | O | 85 |
|  | P2 | 162 | 8 | 210 | 150 | 3 | O | 85 |
|  | P3 | 141 | 22 | 171 | 141 | 4 | R | 50 |
|  | P4 | 153 | 19 | 180 | 237 | 4 | OY | 60 |
|  | P5 | 126 | 8 | 141 | 165 | 2 | O | 45 |
|  | P6 | 129 | 3.5 | 90 | 90 | 1 | R | 30 |
|  | P7 | 300 | 10 | 258 | 330 | 1 | YO | 95 |
|  | P8 | 243 | 12 | 177 | 162 | 3 | R | 60 |
|  | P9 | 195 | 13 | 93 | 165 | 9 | P | 65 |
|  | P10 | 255 | 12 | 246 | 183 | 3 | R | 80 |
|  | P11 | 144 | 6.5 | 93 | 165 | 3 | OY | 70 |
|  | P12 | 126 | 7 | 195 | 219 | 1 | OY | 70 |
|  | P13 | 144 | 22 | 210 | 210 | 8 | PY | 70 |
|  | P14 | 177 | 13.5 | 129 | 162 | 4 | OY | 75 |
| Gulmit | G1 | 228 | 5 | 195 | 177 | 1 | R | 40 |
|  | G2 | 216 | 6 | 126 | 138 | 1 | OR | 50 |
|  | G3 | 210 | 23 | 219 | 180 | 9 | OR | 65 |
|  | G4 | 180 | 5 | 81 | 60 | 1 | OR | 60 |
|  | G5 | 216 | 12 | 150 | 153 | 3 | OR | 60 |
|  | G6 | 243 | 8 | 228 | 186 | 1 | Y | 60 |
|  | G7 | 141 | 6.5 | 159 | 159 | 1 | PY | 20 |
|  | G8 | 285 | 21 | 210 | 195 | 4 | R | 35 |
|  | G9 | 153 | 5.5 | 141 | 138 | 1 | OY | 50 |
|  | G10 | 195 | 6 | 174 | 180 | 1 | OY | 18 |
|  | G11 | 189 | 13 | 255 | 150 | 2 | OY | 40 |
|  | G12 | 297 | 21 | 156 | 279 | 2 | PY | 55 |
|  | G13 | 186 | 7 | 183 | 180 | 2 | OY | 65 |
|  | G14 | 255 | 6 | 165 | 165 | 1 | O | 58 |
| Shiskat | Sh1 | 234 | 4 | 168 | 132 | 1 | OY | 65 |
|  | Sh2 | 210 | 9 | 147 | 174 | 1 | PY | 35 |
|  | Sh3 | 207 | 16 | 195 | 207 | 4 | PY | 30 |
|  | Sh4 | 300 | 11 | 210 | 150 | 1 | OR | 10 |
|  | Sh5 | 495 | 24 | 450 | 480 | 1 | R | 20 |
|  | Sh6 | 411 | 11 | 309 | 345 | 1 | OR | 75 |
|  | Sh7 | 168 | 32 | 186 | 135 | 6 | OR | 75 |
|  | Sh8 | 240 | 6 | 189 | 168 | 1 | Y | 40 |
|  | Sh9 | 315 | 12 | 183 | 150 | 2 | OY | 30 |
|  | Sh10 | 255 | 9.5 | 225 | 228 | 2 | PY | 60 |
|  | Sh11 | 213 | 9 | 225 | 225 | 1 | R | 27 |
|  | Sh12 | 255 | 9 | 201 | 243 | 1 | OR | 55 |
|  | Sh13 | 255 | 11 | 240 | 270 | 1 | OR | 70 |
|  | Sh14 | 210 | 4 | 189 | 159 | 1 | OR | 40 |

GBH: Girth at Breast Height, WE: West-East, NS: North-South, O: Orange, Y: Yellow, P: Pale, R: Red

Table S2: Gene IDs of all species including *Arabidopsis thaliana* (*AtAKR1-22*), *Oryza sativa* (*OsAKR1-33*), *Chlamydomonas rhamnoides* (*CrAKR1-19*), *Fragaria ananasa (FaAKR01-102*)

| Gene Identifier | Transcript ID |
| --- | --- |
| *HrAKR01* | Sph_Contig03961_ERROPOS3400000G001240 |
| *HrAKR02* | Sph_LG0G001140 |
| *HrAKR03* | Sph_LG10G001485 |
| *HrAKR04* | Sph_LG10G002184 |
| *HrAKR05* | Sph_LG11G000058 |
| *HrAKR06* | Sph_LG11G000227 |
| *HrAKR07* | Sph_LG11G002201 |
| *HrAKR08* | Sph_LG11G002282 |
| *HrAKR09* | Sph_LG11G002378 |
| *HrAKR10* | Sph_LG11G004027 |
| *HrAKR11* | Sph_LG2G000800 |
| *HrAKR12* | Sph_LG3G000711 |
| *HrAKR13* | Sph_LG4G000408 |
| *HrAKR14* | Sph_LG6G001241 |
| *HrAKR15* | Sph_LG7G000377 |
| *HrAKR16* | Sph_LG9G000577 |
| *HrAKR17* | Sph_LG9G001884 |
| *HrAKR18* | Sph_LG9G003405 |
| *AtAKR01* | AT2G37770\|AT2G37770.2 |
| *AtAKR02* | AT2G21260\|AT2G21260.1 |
| *AtAKR03* | AT2G37790\|AT2G37790.1 |
| *AtAKR04* | AT2G37760\|AT2G37760.1 |
| *AtAKR05* | AT2G27680\|AT2G27680.1 |
| *AtAKR06* | AT2G21250\|AT2G21250.1 |
| *AtAKR07* | AT4G33670\|AT4G33670.1 |
| *AtAKR08* | AT1G60710\|AT1G60710.1 |
| *AtAKR09* | AT1G06690\|AT1G06690.1 |
| *AtAKR10* | AT1G04420\|AT1G04420.1 |
| *AtAKR11* | AT1G10810\|AT1G10810.1 |
| *AtAKR12* | AT1G59960\|AT1G59960.1 |
| *AtAKR13* | AT1G60730\|AT1G60730.3 |
| *AtAKR14* | AT1G60750\|AT1G60750.1 |
| *AtAKR15* | AT1G04690\|AT1G04690.1 |
| *AtAKR16* | AT1G60690\|AT1G60690.1 |
| *AtAKR17* | AT1G60680\|AT1G60680.1 |
| *AtAKR18* | AT1G59950\|AT1G59950.1 |
| *AtAKR19* | AT3G53880\|AT3G53880.1 |
| *AtAKR20* | AT5G62420\|AT5G62420.1 |
| *AtAKR21* | AT5G53580\|AT5G53580.1 |
| *AtAKR22* | AT5G01670\|AT5G01670.2 |
| *OsAKR01* | LOC_Os10g02470\|LOC_Os10g02470.1 |
| *OsAKR02* | LOC_Os10g02490\|LOC_Os10g02490.2 |
| *OsAKR03* | LOC_Os10g02480\|LOC_Os10g02480.1 |
| *OsAKR04* | LOC_Os10g28320\|LOC_Os10g28320.1 |
| *OsAKR05* | LOC_Os10g02380\|LOC_Os10g02380.1 |
| *OsAKR06* | LOC_Os10g37330\|LOC_Os10g37330.1 |
| *OsAKR07* | LOC_Os04g26910\|LOC_Os04g26910.1 |
| *OsAKR08* | LOC_Os04g27060\|LOC_Os04g27060.1 |
| *OsAKR09* | LOC_Os04g08550\|LOC_Os04g08550.1 |
| *OsAKR10* | LOC_Os04g26920\|LOC_Os04g26920.3 |
| *OsAKR11* | LOC_Os04g37490\|LOC_Os04g37490.1 |
| *OsAKR12* | LOC_Os04g37480\|LOC_Os04g37480.1 |
| *OsAKR13* | LOC_Os04g26870\|LOC_Os04g26870.1 |
| *OsAKR14* | LOC_Os04g37470\|LOC_Os04g37470.1 |
| *OsAKR15* | LOC_Os04g26890\|LOC_Os04g26890.1 |
| *OsAKR16* | LOC_Os04g27300\|LOC_Os04g27300.1 |
| *OsAKR17* | LOC_Os07g05000\|LOC_Os07g05000.1 |
| *OsAKR18* | LOC_Os07g04990\|LOC_Os07g04990.1 |
| *OsAKR19* | LOC_Os01g62870\|LOC_Os01g62870.1 |
| *OsAKR20* | LOC_Os01g47380\|LOC_Os01g47380.1 |
| *OsAKR21* | LOC_Os01g62860\|LOC_Os01g62860.1 |
| *OsAKR22* | LOC_Os01g62880\|LOC_Os01g62880.1 |
| *OsAKR23* | LOC_Os01g43090\|LOC_Os01g43090.1 |
| *OsAKR24* | LOC_Os03g41510\|LOC_Os03g41510.1 |
| *OsAKR25* | LOC_Os03g13390\|LOC_Os03g13390.2 |
| *OsAKR26* | LOC_Os02g03100\|LOC_Os02g03100.1 |
| *OsAKR27* | LOC_Os02g57240\|LOC_Os02g57240.1 |
| *OsAKR28* | LOC_Os09g39390\|LOC_Os09g39390.1 |
| *OsAKR29* | LOC_Os12g29760\|LOC_Os12g29760.1 |
| *OsAKR30* | LOC_Os05g38230\|LOC_Os05g38230.1 |
| *OsAKR31* | LOC_Os05g39690\|LOC_Os05g39690.1 |
| *OsAKR32* | LOC_Os11g42540\|LOC_Os11g42540.1 |
| *OsAKR33* | LOC_Os11g32270\|LOC_Os11g32270.1 |
| *CreAKR01* | Cre11.g467622\|Cre11.g467622.t1.1 |
| *CreAKR02* | Cre14.g630400\|Cre14.g630400.t1.2 |
| *CreAKR03* | Cre16.g684750\|Cre16.g684750.t1.2 |
| *CreAKR04* | Cre16.g692800\|Cre16.g692800.t1.1 |
| *CreAKR05* | Cre06.g294450\|Cre06.g294450.t1.1 |
| *CreAKR06* | Cre06.g276050\|Cre06.g276050.t1.2 |
| *CreAKR07* | Cre09.g394658\|Cre09.g394658.t1.1 |
| *CreAKR08* | Cre17.g699900\|Cre17.g699900.t1.2 |
| *CreAKR09* | Cre17.g730700\|Cre17.g730700.t1.1 |
| *CreAKR10* | Cre02.g095127\|Cre02.g095127.t1.1 |
| *CreAKR11* | Cre03.g201327\|Cre03.g201327.t1.1 |
| *CreAKR12* | Cre03.g195050\|Cre03.g195050.t1.2 |
| *CreAKR13* | Cre10.g432900\|Cre10.g432900.t1.2 |
| *CreAKR14* | Cre10.g461900\|Cre10.g461900.t1.2 |
| *CreAKR15* | Cre04.g218500\|Cre04.g218500.t1.2 |
| *CreAKR16* | Cre04.g217916\|Cre04.g217916.t1.1 |
| *CreAKR17* | Cre04.g216350\|Cre04.g216350.t1.2 |
| *CreAKR18* | Cre12.g518900\|Cre12.g518900.t1.2 |
| *CreAKR19* | Cre12.g544000\|Cre12.g544000.t1.2 |
| *FaAKR01* | augustus_masked-Fvb5-4-processed-gene-82.0 |
| *FaAKR02* | maker-Fvb5-3-augustus-gene-178.32 |
| *FaAKR03* | maker-Fvb6-2-snap-gene-196.46 |
| *FaAKR04* | maker-Fvb6-2-augustus-gene-258.74 |
| *FaAKR05* | maker-Fvb6-2-snap-gene-197.48 |
| *FaAKR06* | maker-Fvb6-2-snap-gene-258.98 |
| *FaAKR07* | maker-Fvb6-2-augustus-gene-197.37 |
| *FaAKR08* | maker-Fvb6-2-snap-gene-258.96 |
| *FaAKR09* | snap_masked-Fvb6-2-processed-gene-197.13 |
| *FaAKR10* | maker-Fvb6-2-augustus-gene-356.30 |
| *FaAKR11* | maker-Fvb6-2-snap-gene-197.49 |
| *FaAKR12* | maker-Fvb4-3-augustus-gene-8.40 |
| *FaAKR13* | maker-Fvb4-3-augustus-gene-295.47 |
| *FaAKR14* | maker-Fvb4-3-augustus-gene-310.40 |
| *FaAKR15* | maker-Fvb4-3-snap-gene-4.66 |
| *FaAKR16* | maker-Fvb4-3-augustus-gene-8.39 |
| *FaAKR17* | maker-Fvb4-3-augustus-gene-278.15 |
| *FaAKR18* | augustus_masked-Fvb7-3-processed-gene-44.10 |
| *FaAKR19* | maker-Fvb7-3-augustus-gene-43.53 |
| *FaAKR20* | maker-Fvb7-3-augustus-gene-18.53 |
| *FaAKR21* | maker-Fvb3-2-augustus-gene-103.28 |
| *FaAKR22* | snap_masked-Fvb3-2-processed-gene-200.24 |
| *FaAKR23* | maker-Fvb7-1-augustus-gene-288.56 |
| *FaAKR24* | maker-Fvb7-1-augustus-gene-274.53 |
| *FaAKR25* | maker-Fvb7-1-augustus-gene-315.58 |
| *FaAKR26* | maker-Fvb7-1-snap-gene-299.78 |
| *FaAKR27* | maker-Fvb7-2-augustus-gene-262.58 |
| *FaAKR28* | maker-Fvb7-2-augustus-gene-259.62 |
| *FaAKR29* | maker-Fvb5-1-augustus-gene-110.33 |
| *FaAKR30* | maker-Fvb6-3-snap-gene-406.72 |
| *FaAKR31* | snap_masked-Fvb6-3-processed-gene-402.50 |
| *FaAKR32* | maker-Fvb6-3-snap-gene-399.94 |
| *FaAKR33* | maker-Fvb6-3-snap-gene-402.99 |
| *FaAKR34* | maker-Fvb6-3-snap-gene-338.48 |
| *FaAKR35* | maker-Fvb6-3-snap-gene-407.72 |
| *FaAKR36* | maker-Fvb6-3-snap-gene-338.42 |
| *FaAKR37* | snap_masked-Fvb6-3-processed-gene-419.40 |
| *FaAKR38* | maker-Fvb6-3-snap-gene-419.81 |
| *FaAKR39* | maker-Fvb6-3-augustus-gene-402.85 |
| *FaAKR40* | snap_masked-Fvb6-3-processed-gene-419.34 |
| *FaAKR41* | maker-Fvb6-3-snap-gene-419.86 |
| *FaAKR42* | maker-Fvb6-3-augustus-gene-338.32 |
| *FaAKR43* | maker-Fvb2-4-augustus-gene-181.35 |
| *FaAKR44* | maker-Fvb2-4-augustus-gene-3.21 |
| *FaAKR45* | maker-Fvb2-4-augustus-gene-9.45 |
| *FaAKR46* | maker-Fvb2-4-snap-gene-1.42 |
| *FaAKR47* | maker-Fvb2-4-snap-gene-78.43 |
| *FaAKR48* | maker-Fvb2-1-augustus-gene-7.38 |
| *FaAKR49* | maker-Fvb2-1-snap-gene-159.62 |
| *FaAKR50* | augustus_masked-Fvb2-1-processed-gene-179.16 |
| *FaAKR51* | maker-Fvb6-4-snap-gene-36.67 |
| *FaAKR52* | maker-Fvb6-4-snap-gene-94.32 |
| *FaAKR53* | augustus_masked-Fvb6-4-processed-gene-289.0 |
| *FaAKR54* | augustus_masked-Fvb6-4-processed-gene-94.0 |
| *FaAKR55* | snap_masked-Fvb6-4-processed-gene-36.29 |
| *FaAKR56* | snap_masked-Fvb6-4-processed-gene-36.28 |
| *FaAKR57* | maker-Fvb6-4-augustus-gene-94.25 |
| *FaAKR58* | maker-Fvb6-1-augustus-gene-343.59 |
| *FaAKR59* | maker-Fvb6-1-augustus-gene-85.45 |
| *FaAKR60* | snap_masked-Fvb6-1-processed-gene-343.40 |
| *FaAKR61* | snap_masked-Fvb6-1-processed-gene-274.24 |
| *FaAKR62* | snap_masked-Fvb6-1-processed-gene-343.36 |
| *FaAKR63* | maker-Fvb6-1-augustus-gene-274.46 |
| *FaAKR64* | augustus_masked-Fvb6-1-processed-gene-343.15 |
| *FaAKR65* | maker-Fvb6-1-augustus-gene-274.43 |
| *FaAKR66* | maker-Fvb6-1-augustus-gene-343.54 |
| *FaAKR67* | maker-Fvb6-1-snap-gene-274.55 |
| *FaAKR68* | maker-Fvb4-2-augustus-gene-5.52 |
| *FaAKR69* | maker-Fvb4-2-snap-gene-8.66 |
| *FaAKR70* | maker-Fvb4-2-augustus-gene-230.22 |
| *FaAKR71* | snap_masked-Fvb4-2-processed-gene-141.17 |
| *FaAKR72* | maker-Fvb4-2-snap-gene-248.40 |
| *FaAKR73* | maker-Fvb4-2-augustus-gene-211.31 |
| *FaAKR74* | maker-Fvb4-2-augustus-gene-60.32 |
| *FaAKR75* | augustus_masked-Fvb3-3-processed-gene-178.6 |
| *FaAKR76* | maker-Fvb3-3-augustus-gene-102.31 |
| *FaAKR77* | maker-Fvb3-4-augustus-gene-111.28 |
| *FaAKR78* | maker-Fvb3-4-augustus-gene-186.32 |
| *FaAKR79* | maker-Fvb7-4-augustus-gene-33.48 |
| *FaAKR80* | maker-Fvb7-4-augustus-gene-7.43 |
| *FaAKR81* | maker-Fvb4-4-augustus-gene-6.55 |
| *FaAKR82* | maker-Fvb4-4-snap-gene-59.50 |
| *FaAKR83* | augustus_masked-Fvb4-4-processed-gene-152.13 |
| *FaAKR84* | maker-Fvb4-4-snap-gene-10.69 |
| *FaAKR85* | maker-Fvb4-4-augustus-gene-255.41 |
| *FaAKR86* | maker-Fvb4-1-augustus-gene-24.32 |
| *FaAKR87* | maker-Fvb4-1-snap-gene-196.42 |
| *FaAKR88* | maker-Fvb4-1-augustus-gene-196.31 |
| *FaAKR89* | maker-Fvb4-1-snap-gene-10.45 |
| *FaAKR90* | maker-Fvb4-1-augustus-gene-38.23 |
| *FaAKR91* | maker-Fvb4-1-augustus-gene-199.65 |
| *FaAKR92* | maker-Fvb4-1-augustus-gene-141.33 |
| *FaAKR93* | maker-Fvb2-2-augustus-gene-219.47 |
| *FaAKR94* | maker-Fvb2-2-augustus-gene-225.38 |
| *FaAKR95* | maker-Fvb2-2-augustus-gene-52.66 |
| *FaAKR96* | maker-Fvb2-2-augustus-gene-156.34 |
| *FaAKR97* | maker-Fvb5-2-augustus-gene-103.34 |
| *FaAKR98* | maker-Fvb2-3-augustus-gene-230.24 |
| *FaAKR99* | maker-Fvb2-3-snap-gene-89.40 |
| *FaAKR100* | snap_masked-Fvb2-3-processed-gene-89.22 |
| *FaAKR101* | maker-Fvb2-3-snap-gene-158.34 |
| *FaAKR102* | snap_masked-Fvb2-3-processed-gene-222.21 |

Table S3. List of primers used in the study

| Gene code | Transcript ID | CDS | Forward Primer | Reverse Primer |
| --- | --- | --- | --- | --- |
| HrAKR01 | Sph_Contig03961_ERROPOS3400000G001240 | 1053 | CATGGATGCACTCCTGTACAGC | GATTGGCCATCATTTGGTGGTG |
| HrAKR02 | Sph_LG0G001140 | 366 | GCATGCCTTCCTTCTCAACTAG | GGTGGAGTGTCGGAAAATTTCC |
| HrAKR03 | Sph_LG10G001485 | 930 | ATGTTTGGATGATCCAGCTCTC | TTAAGCATAGAGATCTACGCCC |
| HrAKR04 | Sph_LG10G002184 | 1356 | TTCTGAAGGAGAATCCAGATAC | GAATGGGTGGGTGGGTAACTG |
| HrAKR05 | Sph_LG11G000058 | 1101 | GACAGCAAACTATGCTCCTCTGC | GCCAAGAGAATCACATATGTTC |
| HrAKR06 | Sph_LG11G000227 | 951 | TTGATTTTGCCTCTGTTCGTCC | GCAACCTGAGCAGGAGTAGC |
| HrAKR07 | Sph_LG11G002201 | 948 | CTGGATGTGGCACGTGTTCC | AACGAAGAGCAACCTGTGCAG |
| HrAKR08 | Sph_LG11G002282 | 747 | GAGTATTGCAAGAACAATGCC | GATTTCCCAGCCAAAAACCTG |
| HrAKR09 | Sph_LG11G002378 | 936 | CTTTGCCGAGAGCTTGGTATC | ACTTTTGTTGTCCCTGGGATTG |
| HrAKR10 | Sph_LG11G004027 | 1272 | AGTGTAGAGAAAAGCCTTAAGCG | CCATATGAAGTCTCATTGGAGAC |
| HrAKR11 | Sph_LG2G000800 | 957 | GGACTAAATGGGGAGGTGATA | CTGAGATCTTCCTCAGTCAAT |
| HrAKR12 | Sph_LG3G000711 | 1155 | ATGGCAGAGCTTTGTGAGCTC | CTGAAGCAGGGCTTGGAACAG |
| HrAKR13 | Sph_LG4G000408 | 1080 | CGCTCGGAGTACCTATTTCAAC | CTACATGGCAGGACACTAGTTC |
| HrAKR14 | Sph_LG6G001241 | 1101 | ATTCACCTATTGGTCAAGGTGC | CAGCCTGTTCCGCATTTTTAGC |
| HrAKR15 | Sph_LG7G000377 | 987 | TCGCTCACTTGTTGACGATGTG | GGCTTGGTTTGTATAACAGCTT |
| HrAKR16 | Sph_LG9G000577 | 897 | CAAGTGGAATGCCATCCTGTTTG | GGAAGGATACTATGTCCACTTTG |
| HrAKR17 | Sph_LG9G001884 | 1008 | ATAACAAGGGCGATATACCACC | AATCTGTGACTCCTTTGTAGCC |
| HrAKR18 | Sph_LG9G003405 | 972 | ATGGGACTTCTTACTGAAAGTGG | CAGTAGCATTCTCTTCAACCTGT |
| HrUBQ | Sph_LG2G000372 | 816 | AGGGCTTCAGAAGATGAGCTGA | ATGCCATCCTGATCCCACTCTG |

**Table S4: Analysis of variance for the fruit quality traits in seventy sea-buckthorn accessions**

| SOV | Df | Fe | Zn | FC | FS | FL | FD | FV | FM | FW | DW |
| --- | --- | --- | --- | --- | --- | --- | --- | --- | --- | --- | --- |
| Replications | 2 | 15.5 | 0.7 | 1.2 | 88.0 | 0.1 | 0.0 | 0.1 | 19.7. | 1.03 | 0.01 |
| Accessions | 69 | 15342.9*** | 31.9*** | 11.4*** | 610.4*** | 2.5*** | 1.6*** | 3.08*** | 34.1*** | 1.0*** | 0.02*** |
| Residuals | 138 | 155.8 | 0.3 | 1.2 | 110.7 | 0.1 | 0.1 | 0.3 | 7.2 | 0.03 | 0.00 |

Significance codes: 0.001 ‘***’, 0.01 ‘**’, 0.05 ‘*’, 0.1 ‘.’

SOV: source of variation, Df: degree of freedom, Fe: iron, Zn: zinc, FC: fruit colour, FS: fruit setting percentage, FM: fruit moisture, FW: fresh weight of fruit, DW: dry weight of fruit, FV: fruit volume, FL: fruit length, FD: fruit diamter.


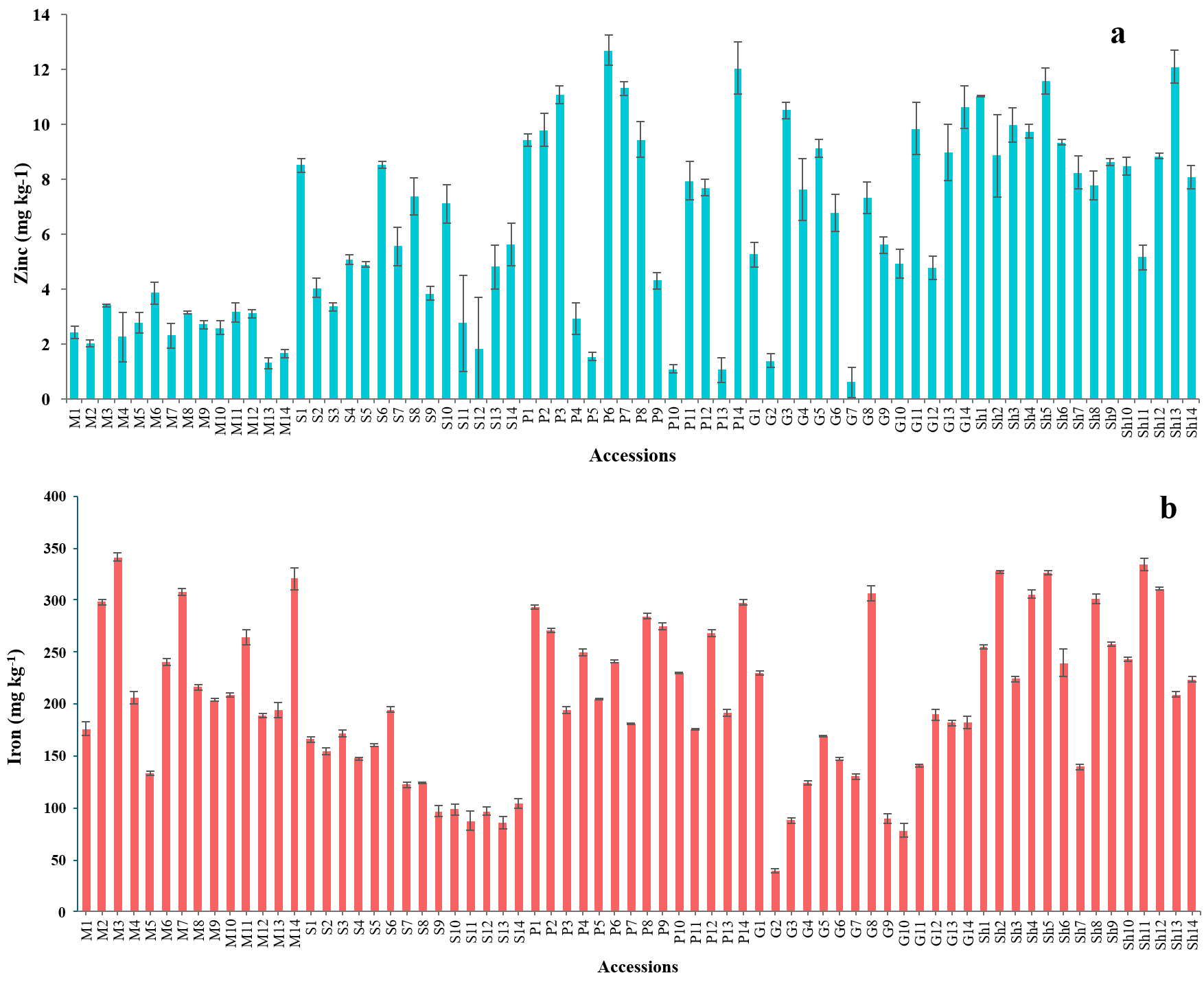


Figure S1. Micronutrient content in seventy accessions of sea-buckthorn from different altitudes.

a. Iron content b. Zinc content. M: Misgar, S: Sost, P: Passu, G: Gulmit, Sh: Shishkat. The black bars were drawn based on standard error.

**Figure S2.** Frequency distribution of *HrAKRs* across sub-cellular organelles.

Cyto: cytoplasm, Nucl: nucleus, Chloro: chloroplast, Mito: mitochondria, extra: extracellular, Vac: vacuole, E.R: endoplasmic reticulum, Golg: golgi bodies, Cysk: cytoskeleton, Plas: plasma membrane


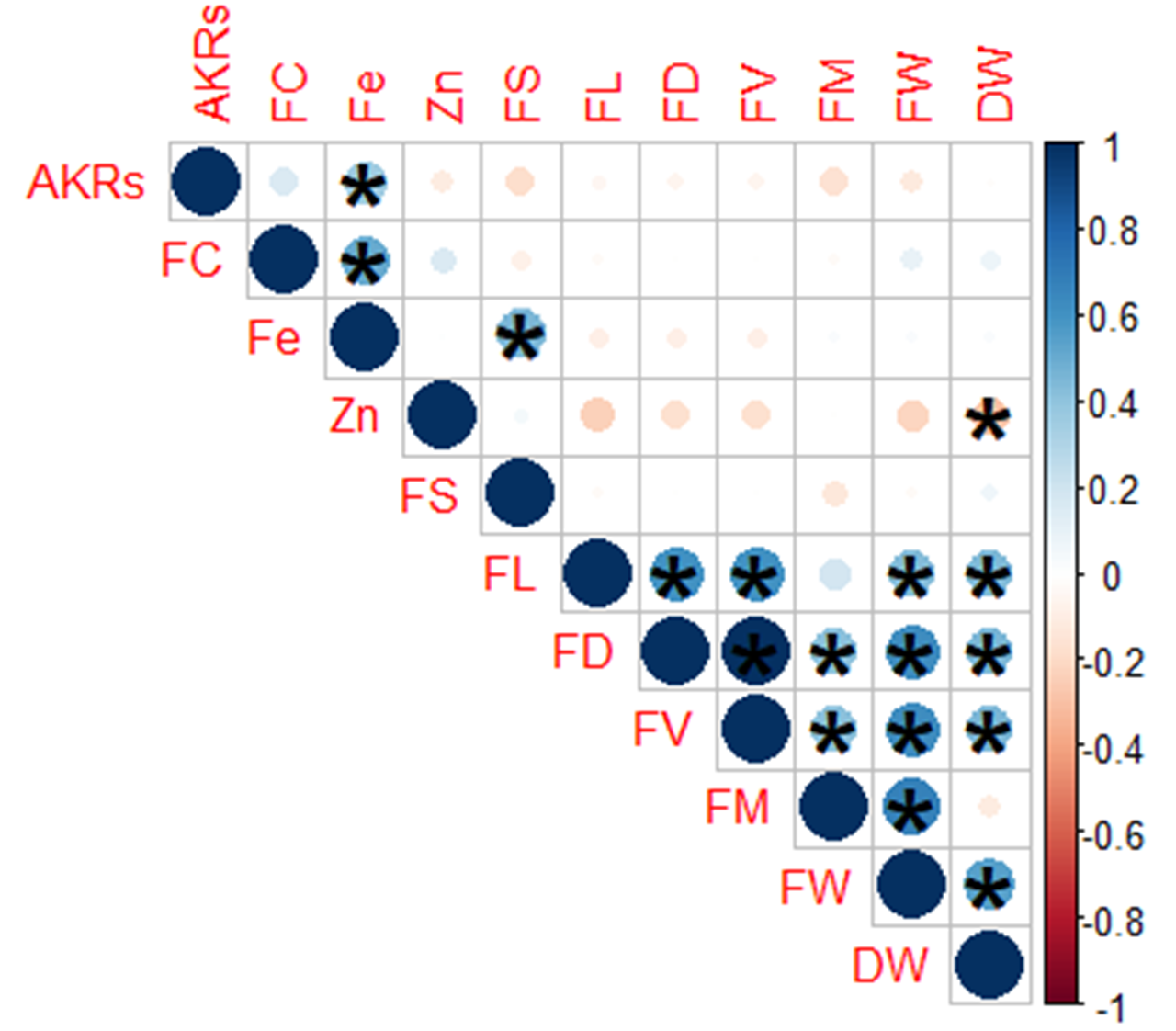
Figure S3: Correlation plot for the morphological traits, iron, zinc, and number of transcripts expressed in seventy seabuckthorn accessions.

Table S5. Physio-chemical properties and sub-cellular localization of HrAKR proteins

| Gene Name | Gene ID | Gen | CDS | AA | MW Kda | pI | GRAVY | Localization |
| --- | --- | --- | --- | --- | --- | --- | --- | --- |
| HrAKR01 | Sph_Contig03961 | 1451 | 1053 | 350 | 39053.68 | 5.49 | -0.2 | C |
| HrAKR02 | Sph_LG0G001140 | 366 | 366 | 121 | 13426.54 | 5.62 | -0.107 | E, N, C |
| HrAKR03 | Sph_LG10G001485 | 1378 | 930 | 309 | 34801.98 | 6.02 | -0.183 | C |
| HrAKR04 | Sph_LG10G002184 | 2275 | 1356 | 451 | 50560.35 | 8.29 | -0.013 | PM |
| HrAKR05 | Sph_LG11G000058 | 1773 | 1101 | 366 | 41019.57 | 9.44 | -0.177 | M, C |
| HrAKR06 | Sph_LG11G000227 | 951 | 951 | 316 | 35565.63 | 5.39 | -0.268 | C |
| HrAKR07 | Sph_LG11G002201 | 1388 | 948 | 315 | 35292.48 | 6.49 | -0.238 | C |
| HrAKR08 | Sph_LG11G002282 | 747 | 747 | 249 | 28066.01 | 7.28 | -0.299 | M |
| HrAKR09 | Sph_LG11G002378 | 936 | 936 | 311 | 34835.08 | 6.45 | -0.246 | C |
| HrAKR10 | Sph_LG11G004027 | 1559 | 1272 | 424 | 47472.97 | 8.16 | -0.34 | C, M |
| HrAKR11 | Sph_LG2G000800 | 1098 | 957 | 318 | 35543.26 | 6.29 | -0.134 | C |
| HrAKR12 | Sph_LG3G000711 | 1538 | 1155 | 384 | 43443.18 | 8.85 | -0.258 | N, C, M |
| HrAKR13 | Sph_LG4G000408 | 2044 | 1080 | 359 | 39594.41 | 8.4 | -0.174 | C, Ch |
| HrAKR14 | Sph_LG6G001241 | 1665 | 1101 | 366 | 40158.83 | 8.6 | -0.202 | Ch |
| HrAKR15 | Sph_LG7G000377 | 1453 | 987 | 328 | 36576.93 | 6.52 | -0.216 | C, Ch |
| HrAKR16 | Sph_LG9G000577 | 1120 | 897 | 298 | 32936.05 | 8.16 | -0.104 | C |
| HrAKR17 | Sph_LG9G001884 | 1796 | 1008 | 335 | 37515.69 | 5.94 | -0.345 | C, Ch |
| HrAKR18 | Sph_LG9G003405 | 1613 | 972 | 323 | 34848.92 | 5.54 | -0.061 | C, Ch |

**Table S6.** In silico prediction of subcellular localization signals of proteins generated through NLSdb (Nuclear Localization Signal database)

| Query | Signal | Signal Type | Start | End | Conf Nuc | Conf Fam | Annotation Type | Origin |
| --- | --- | --- | --- | --- | --- | --- | --- | --- |
| HrAKR12 | KRIRPR | NLS | 28 | 33 | 2 | 2 | Potential | In Silico Mutagenesis |
| HrAKR13 | LIDLGL | NES | 155 | 160 | 2 | 2 | Potential | In Silico Mutagenesis |

**Table S7: Nested analysis of variance for the fruit quality traits across five locations**

| SOV | Df | Fe | Zn | FC | FS | FL | FD | FV | FM | FW | DW |
| --- | --- | --- | --- | --- | --- | --- | --- | --- | --- | --- | --- |
| Location | 4 | 139054*** | 93.9*** | 21.2*** | 1813.8*** | 1.8*** | 1.1*** | 2.0*** | 99.1*** | 0.5*** | 0.1*** |
| Location: Accessions | 65 | 7730*** | 28.0*** | 10.8*** | 589.8*** | 2.5*** | 1.6*** | 3.1*** | 30.1*** | 0.9*** | 0.01*** |
| Location: Replication | 10 | 179 | 0.5 | 0.8 | 280* | 0.2 | 0.1 | 0.2 | 9.35 | 0.1 | 0.003 |
| Residuals | 130 | 152 | 0.3 | 1.2 | 63.5 | 0.1 | 0.1 | 0.3 | 7.19 | 0.1 | 0.003 |

Significance codes: 0.001 ‘***’, 0.01 ‘**’, 0.05 ‘*’, 0.1 ‘.’

SOV: source of variation, Df: degree of freedom, Fe: iron, Zn: zinc, FC: fruit colour, FS: fruit setting percentage, FL: fruit length, FD: fruit diamter FV: fruit volume, FM: fruit moisture, FW: fresh weight of fruit, DW: dry weight of fruit

**PCR reaction mixture, amplification conditions, and gel electrophoresis details**

PCR reactions were performed in a 15 μl reaction mixture containing: 2 μl 10×PCR buffer, 1.6 μl (SS) SgCl_2_, 1 μl 10SS dNTPs, 1 μl each of 0.5 μM forward and reverse primers, 0.2 μl of Taq DNA polymerase (1 U), 1μl template DNA and 7.2 μl ultra-pure d_2_H_2_O.

Amplification conditions included: an initial denaturation at 94 °C for 4Sin followed by denaturation at 94 °C for 30 s, annealing at 55-62 °C for 30 s and extension at 68 °C for 30 s, these steps were repeated 39 times before the final extension at 68 °C for 10 Sin.

Seven µl of each PCR product was fractionated in a 2% agarose gel and electrophoresed in 1× TAE buffer at 90V for 20 Sin. The gel was stained with 3 µl ethidium bromide and visualized under UV light using an OmegaFlour Plus documentation system (Omega Flour Plus Inc., San Francisco, CA 94107 USA).
